# Sexual Health in Child and Adolescent Psychiatry: Multi-Site Implementation Through Synchronized Videoconferencing of an Educational Resource Using Standardized Patients

**DOI:** 10.3389/fpsyt.2020.593101

**Published:** 2020-11-17

**Authors:** Linda Drozdowicz, Elisabeth Gordon, Desiree Shapiro, Sansea Jacobson, Isheeta Zalpuri, Colin Stewart, A. Lee Lewis, Lee Robinson, Myo Thwin Myint, Peter Daniolos, Edwin D. Williamson, Richard Pleak, Ana Soledade Graeff Martins, Mary Margaret Gleason, Cathryn A. Galanter, Sarah Miller, Dorothy Stubbe, Andrés Martin

**Affiliations:** ^1^Child Study Center, Yale School of Medicine, New Haven, CT, United States; ^2^Private Practice, New York, NY, United States; ^3^University of California, San Diego, San Diego, CA, United States; ^4^Western Psychiatric Hospital, Pittsburgh, PA, United States; ^5^Stanford University, Palo Alto, CA, United States; ^6^Georgetown University, Washington, DC, United States; ^7^Medical University of South Carolina, Charleston, SC, United States; ^8^Cambridge Health Alliance, Cambridge, MA, United States; ^9^Tulane University, New Orleans, LA, United States; ^10^University of Iowa, Iowa City, IA, United States; ^11^Vanderbilt University Medical Center, Nashville, TN, United States; ^12^Zucker School of Medicine at Hofstra/Northwell, Hempstead, NY, United States; ^13^Federal University of Rio Grande do Sul, Porto Alegre, Brazil; ^14^Children's Hospital of the King's Daughters in Virginia, Norfolk, VA, United States; ^15^SUNY Downstate Medical Center, Brooklyn, NY, United States; ^16^Kings County Hospital Center, Brooklyn, NY, United States; ^17^Temple University, Philadelphia, PA, United States; ^18^Standardized Patient Program, Teaching and Learning Center, Yale School of Medicine, New Haven, CT, United States

**Keywords:** child and adolescence psychiatry, sexual education, standardized patient, simulation, training and education

## Abstract

**Objective:** Matters of sexuality and sexual health are common in the practice of child and adolescent psychiatry (CAP), yet clinicians can feel ill-equipped to address them with confidence. To address this gap in training and practice, we developed, implemented, and evaluated an educational module enhanced by videotaped depictions of expert clinicians interacting with professional actors performing as standardized patients (SPs).

**Methods:** We developed an educational resource highlighting common issues of sexual health relevant to CAP practice, including sexual development, psychotropic-related side effects, and sexuality in children with autism. We wrote original scripts, based on which two clinicians interacted with three SPs. Digital recordings were edited to yield 5 clips with a cumulative running time of 20 min. The clips were interspersed during a 90-min session comprising didactic and interactive components. Due to the COVID-19 pandemic, we used synchronous videoconferencing, which allowed content dissemination to several training programs across the country.

**Results:** We recruited 125 learners from 16 CAP training programs through the American Academy of CAP's Alliance for Learning and Innovation (AALI). Routine inquiry into adolescent patients' sexual function was uncommon, reported by only 28% of participants, with “awkward” and “uncomfortable” the most common terms mentioned in reference to the clinical task. The didactic intervention led to measurable improvements after 2 weeks in skills and knowledge (*p* = 0.004) and in attitudes (*p* < 0.001). The three items with the greatest improvement were: (a) availability of developmentally tailored resources; (b) comfort in addressing sexual development with underage patients; and (c) with parents or guardians of neuroatypical or developmentally disabled patients (*p* < 0.001 for each).

**Conclusions:** A sexual health curriculum enriched by video-based examples can lead to measurable improvement in outcomes pertinent to the clinical practice of CAP. These educational materials are available for distribution, use and adaptation by local instructors. Our study also provides proof-of-principle for the use of multisite educational initiatives in CAP through synchronized videoconferencing.

## Introduction

Sexuality is elemental to the human experience and is engaged in throughout life. Sexual health is a critical element of general health. Defined as a state of physical, emotional, mental, and social wellbeing in relation to sexuality, it is not merely the absence of disease, dysfunction, or infirmity. It requires a positive and respectful approach to sexuality and sexual relationships, as well as the possibility of having pleasurable and safe sexual experiences, free of coercion, discrimination, or violence (1).

The relationship between sexual health and general health is extensive. At the individual level, sexual well-being is positively correlated to physical and mental health, while at the community level, sexual health is associated with improved relationships, economic stability, and increased education and employment (2–6). In particular, sexual health and mental health are bidirectionally influential: Depression is the largest single comorbidity for sexual dysfunction, and sexual dysfunction is associated with a 2.3- to 3.1-fold increased risk for developing a major depressive disorder (7). In the context of the practice of medicine, the relationship between sexual and general health is further complicated by the potential for many medications, including psychotropics, to induce sexual dysfunction (8).

Since sexual health has great potential to impact and be impacted by other aspects of health, physicians should address and support the sexual health of their patients, even as the majority do not (9–11). One of the most significant reason physicians fail at this task is the lack of sexual health education in medical education (9–13), which trainees and physicians are overtly aware of.

Medical sexual health education is an ethical imperative. This education teaches physicians to address sexual health and therefore not cause harm by passively perpetuating the stigma surrounding sexuality (10); to initiate the discussion instead of placing the burden on the patient to do so, which can unintentionally limit access to care around a stigmatized topic (11); and to be aware of and minimize or mitigate iatrogenic sexual dysfunction (12). It also allows physicians to support larger public health efforts by destigmatizing sexuality and addressing it as an important component of health. Without this education, many physicians experience considerable levels of discomfort in addressing sexual health and sexuality with their patients (13–15).

High quality, medically accurate sex education, particularly when started early and provided additively, supports sexual health as children and adolescents develop. It increases empowerment and confidence during a critical period of physical and emotional growth (16) and reduces unprotected sex, pregnancy, and sexually transmitted infections (17). It also improves rates of sexual satisfaction, a key element of sexual health. Yet, many barriers to comprehensive, medically accurate, evidence-based sex education still persist in the US (18). In this context, child and adolescent psychiatrists (CAPs) have an opportunity and duty to intervene and provide relevant sexual health information to patients and families in order to improve their patients' health.

Sex education is often even less accessible to children and adolescents with special needs (19). Reasons range from simply not being provided, to being offered in a way that is inadequate for the individual's learning style or ability (19). This deficiency can have far-reaching effects. In children with autism spectrum disorder (ASD)—a common population in routine CAP practice—good sex education offers benefits beyond those normally expected in the neurotypical population. It provides sexual information directly relevant to physical health, such as how and when to clean one's genitals, menstrual hygiene, and when to seek medical attention—including for sexual symptoms such as loss of libido/desire, erectile dysfunction, or anorgasmia, among others (20). It can help curb the increased risk of sexual victimization for people with ASD (21), and it can also help prevent problematic sexual behaviors, such as public masturbation and unwanted touching, which can have major legal and social ramifications (22).

Moreover, sex education for children and adolescents with ASD or any disability should not be limited to physical health and safety information. Contrary to common assumptions, many people with ASD desire romantic and sexual relationships (23). All children and adolescents deserve and are entitled to the same sexual health education, with the content curated for and presented in a way that is understandable to each individual (23). For those with ASD, this includes concrete teaching about relationships, courtship, and other social skills needed for success—topics that might be picked up from peers in children without ASD, but that may be missed in this population (20).

With this background in mind, we developed, deployed, and evaluated a sexual health educational module specific to the training needs of CAPs. We enhanced our didactic materials with video-based depictions of expert clinicians interacting with professional actors. Due to the COVID-19 pandemic, we provided the training via synchronous videoconferencing, an approach that permitted simultaneous sharing of educational content with several training programs across the country.

## Methods

### Participants and Synchronized Videoconferencing Delivery

Participants comprised CAP faculty, fellows, and residents at Yale and those recruited through the American Academy of Child and Adolescent Psychiatry's Alliance for Learning and Innovation (AALI). Against the backdrop of the COVID-19 pandemic, the didactic was offered exclusively online, using the videoconferencing platform Zoom (San Jose, CA). This approach permitted the course to be offered virtually with synchronous content delivery and real-time interaction between faculty and learners.

### Educational Intervention

We developed the didactic portion of the curriculum under the lead of a psychiatrist with expertise in sex therapy and a CAP with training through NYU's Training Program in Human Sexuality, and with the input of several CAP training directors. We incorporated published recommendations from the Summit on Medical School Education in Sexual Health (24), the needs identified for graduate medical education in sexual health (25), and sexuality matters that are specific to children and adolescents with autism (26–28). For the didactics, we provided basic background education in sexual health, as well as a curated resource list. We focused extensively on those issues of sexual development, health, and management relevant to CAPs. We specifically included information in the didactics on how to address common sexual health issues with patients and their families. The didactics were divided into five parts: (1) Sexual health and sexual development: why it matters and what is normal in childhood; (2) Talking about sexual development in children with autism; (3) Normal sexual development in adolescence, pornography, and “porn literacy;” (4) The management of medication-induced sexual dysfunction; and (5) Special sexuality considerations with autism during adolescence.

To support this content, we developed scripts highlighting common issues of sexuality relevant to CAP, including psychotropic-related side effects and sexuality in children with autism. We made digital recordings of two clinicians interacting with three professional actors, yielding 5 video clips with a cumulative running time of 20 min. The mother in the first scenario, and the adolescent son and his father in the second, are all professional actors hired through the Standardized Patient Program of the Yale School of Medicine's Teaching and Learning Center. The actors are experienced in medical settings and followed standardized patient (SP) best practices (29). The clips outlined in Table 1 were interspersed during a 90-min session comprising didactic and interactive components. We provided a handout with more detail on all didactic components, which also helped accommodate different learning styles. The five video clips and reference handout are included as Supplementary Materials.

**Table 1:** Video clips for child and adolescent psychiatry sexual health didactic resource.

**Clip**	**Scene**	**Content**	**Duration**	
			**Min**	**S**
1	Stephanie and young Hannah	Normal sexuality in children	3	40
2	Stephanie and young Hannah, Part 2	Special considerations in children with autism	5	50
3	Bobby and father	Porn literacy	2	30
4	Bobby	Psychotropic-related sexual side effects	2	0
5	Stephanie and Hannah, three years later	Sexuality and safer sex in adolescents with autism	6	15
Total			20	15

### Outcome Measures

Participants provided demographic information and repeated a survey at baseline and endpoint. At baseline we asked them to provide the first three words or short phrases “that come to mind when discussing sexuality with your adolescent patients” and the three “when discussing sexuality with their parent(s) or guardian(s).” We adapted the *Physician Belief Scale* (30) and the *Patient-Practitioner Orientation Scale* (9) into a 20-item questionnaire tailored to the practice of CAPs. The resulting survey tapped into two common domains in training and education, though is not to be construed as a formal assessment of either: (1) *Skills and Knowledge*, for which respondents indicated how strongly they agree or disagree with 9 statements using a five-point Likert scale; and (2) *Attitudes*, a list of 11 statements similarly coded. Some of the items were reverse-coded to prevent response acquiescence bias.

### Ethics Approval

Before starting data collection, we obtained institutional review board approval from the Yale Human Investigations Committee (Protocol # 2000027918). The study was deemed exempt, with completion of the survey representing tacit consent. Learners were encouraged to participate, but informed that their responses were neither mandatory nor relevant to their performance evaluation. They were notified that results of the surveys would not be accessible to faculty responsible for their assessment. In order to track individuals' responses over time, each participant provided a de-identified and anonymous study code.

### Data Collection and Statistical Analysis

Participants completed surveys through their preferred, WiFi-enabled personal devices during dedicated time on two dates in May and June 2020. We collected information securely through Qualtrics (Provo, UT), and analyzed data using SPSS version 25 (Armonk, NY).

We compared differences before and after the didactic intervention using paired-*t*-tests and Cohen's *d* effect sizes for continuous variables (global scores on Skills and Knowledge, and on Attitudes). We next compared the survey's 20 individual items using chi square-tests for the 20 categorical items, relying on McNemar's-test to examine changes before and after the intervention. McNemar's-test was used to determine differences on a dichotomous dependent variable between two related groups, and is commonly used to analyze pre-post study designs (31). Finally, we used word cloud generator software (wordclouds.com; Zygomatic Inc., Vianen, The Netherlands) to visually depict students' word choices at baseline.

## Results

We approached 20 training programs associated with the AALI network; 15 agreed to participate in one of the dates offered (14 in the US, one in Brazil). Two hundred and nine learners participated in one of the two sessions, and 166 of them (80%) consented to participate and completed the baseline assessment. Some of these learners did not complete the endpoint survey (*n* = 23) or failed to provide a matching unique identifier (*n* = 18). This resulted in a final working sample of 125 participants, yielding a total usable completion rate of 75% (125/143). We compared baseline demographic characteristics between endpoint survey completers (*n* = 125) and non-completers (*n* = 41). Finding no differences (*p* > 0.05 for all contrasts), we restricted analyses to the completer group.

**Figure 1:**
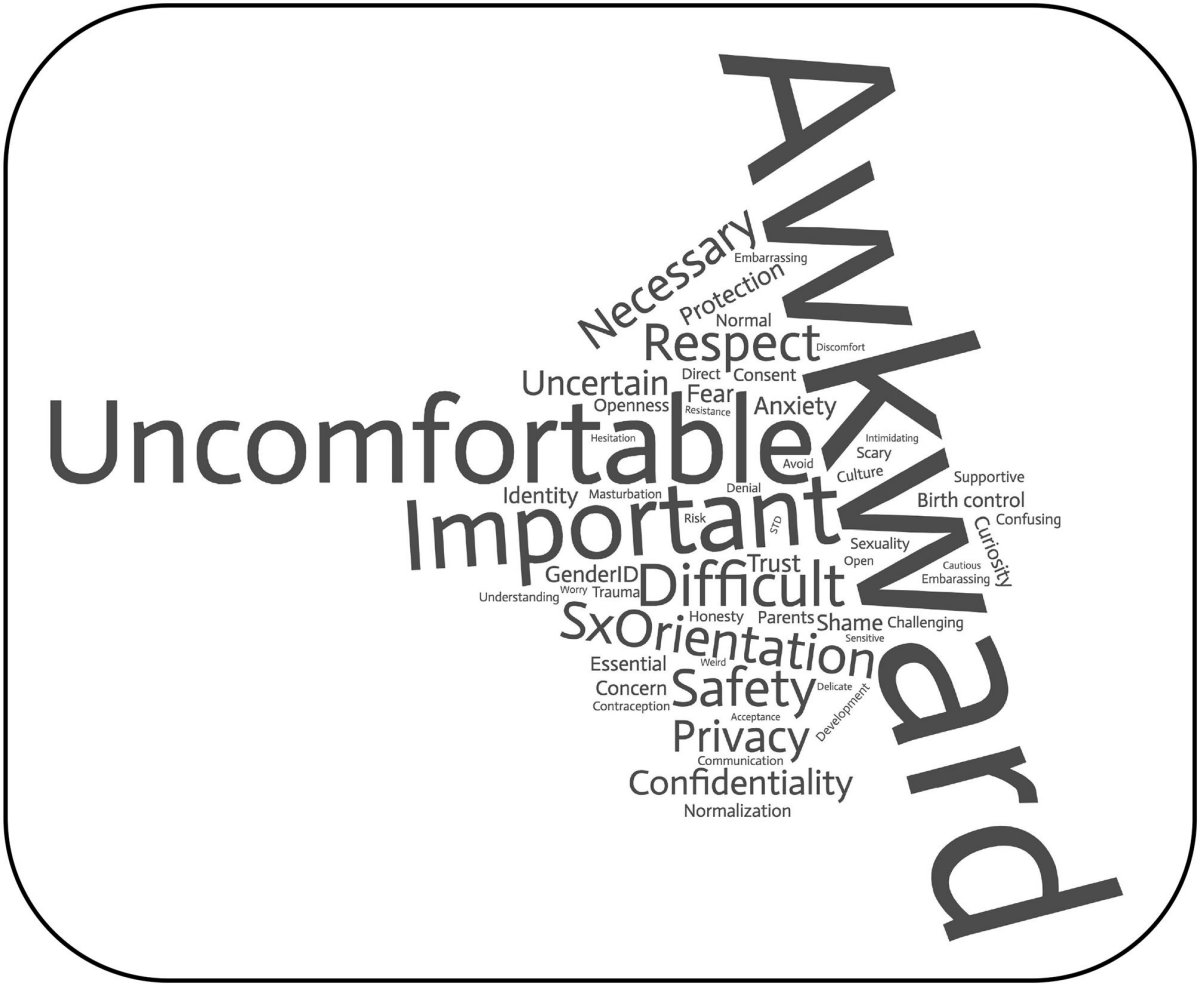
Word cloud based on the prompt “please list words or short phrases that come to mind when discussing sexuality with your adolescent patients [3 words] and/or their parents” [3 words]. *n* = 628 entries, comprising 58 words with 4 or more occurrences each.

Table 2 summarizes characteristics of these 125 participants and their clinical practice routines regarding sexual health. There was a weak correlation between the overall composite score and years in clinical practice (*r* = 0.3, *p* = 0.01), but not with self-rated views on sexuality. Of note, over two thirds of participants acknowledged inquiring about sexual health only “occasionally,” “rarely,” or “never.” Safer sex practices, sexual activity, pregnancy prevention, and sexually transmitted infections were more commonly addressed than masturbation or specific sexual dysfunctions. Addressing patients' sexual activity with parents or guardians was challenging, particularly for patients who are neuro-atypical or have developmental delays. Figure 1 provides a visual depiction of participants' perceptions on addressing sexuality with their patients and guardians, with “awkward” and “uncomfortable” leading the way.

**Table 2:** Descriptive characteristics and clinical practice routines of study participants (*n* = 125).

	***n***	**%**
**Participant characteristics**		
Program size (number of CAP trainees)		
≥10	55	44
8 or 9	34	27
≤7	36	29
Sex		
Female	82	66
Male	43	34
Training level		
Practicing CAP	24	19
Fellow	68	54
Resident	33	26
Continuous variables *(M/SD)*		
Years since MD degree	*6.3*	*6.9*
Self-rated views on sexuality (range, 2–10: lower—more	*7.7*	*1.9*
conservative; higher—more liberal)		
**Clinical practice routines**		
Prompt: *With regard to your child and adolescent patients, how often do you*…^a^		
Inquire about sexual function?	35	28
Inquire about specific sexual behaviors, such as…		
Safer sex practices	72	58
Sexual activity	69	55
Pregnancy prevention	69	55
Intercourse	60	48
Sexually transmitted infections	58	46
Masturbation	11	9
Inquire about changes in sexual function that may be related to psychotropics?	44	35
Inquire about specific sexual problems, such as…		
Desire	13	10
Orgasm	8	6
Arousal (lubrication, erection)	6	5
Refer young patients to specialists in sexual health, as appropriate?	12	10
Discuss sexual health issues with your young patients' parents or guardians?	16	13
Provide education on sexual health to parents or guardians?	14	11
Provide education on sexual side effects of psychotropics to parents or guardians?	41	32

a *Responses refer to number and percentage of participants who responded “Routinely” or “Often” on a given item. Italic values indicate Mean and standard deviation*.

The didactic intervention led to measurable improvements after 2 weeks in Skills and Knowledge (*p* = 0.004), Attitudes (*p* < 0.001), and in the overall composite score (*p* < 0.001; Table 3). Although statistically and clinically significant, effect sizes were small (Cohen's *d* ≤ 0.35). In order to better understand the specific components driving these changes, we conducted a secondary item-level analysis. Table 4 summarizes these findings, rank ordered by the degree of change from baseline to endpoint. The three items with the greatest improvement were: (a) Availability of developmentally tailored resources; (b) Comfort in addressing sexual development with underage patients; and (c) with parents or guardians of neuroatypical or developmentally disabled patients (*p* < 0.001 for each).

**Table 3:** Outcome change after didactic intervention: sexual health skills and attitudes in child and adolescent psychiatry practice (*n* = 125).

**Domain**	**Before**		**After**		**Paired *t* df = 124**	***p***	**Cohen's d**
	**M**	**SD**	**M**	**SD**			
Overall	66.6	7.2	72.1	9.1	8.19	<0.001	0.35
Skills and knowledge	28.0	5.4	31.2	6.0	2.96	0.004	0.24
Attitudes	37.8	4.4	39.1	6.1	6.30	<0.001	0.07

**Table 4:** Item analysis: sexual health skills and attitudes in child and adolescent psychiatry practice (*n* = 125).

**Item**	**Prompt**	**Domain**	**Didactic intervention^*a*^**				
			**Before**		**After**		***p*^*b*^**
			***n***	**%**	***n***	**%**	
1	I have resources to use in support of evaluating, treating and educating young patients regarding their sexual health	S/K	0	0	89	71	<0.001
2	I am comfortable addressing sexual development issues with my underage patients	S/K	61	49	92	74	<0.001
3	I am comfortable addressing sexual development issues with the parents or guardians of my patients (including adults) who are neuro-atypical and/or developmentally disabled	S/K	32	26	70	56	<0.001
4	I approach psychiatric and sexual health problems concurrently	S/K	33	26	54	43	0.001
5	I know the risk of sexual side effects for the medications I prescribe	S/K	33	26	54	43	0.001
6	*Exploring sexual health issues with an underage patient's parents or guardians causes me discomfort	A	35	28	51	41	0.007
7	I address sexual health problems even if only by assessing for their presence and referring to other providers	S/K	38	30	59	47	0.005
8	I recognize when my patients experience a sexual problem	S/K	45	36	63	50	0.01
9	*There are so many issues to be investigated when seeing a patient that I do not always consider sexual health factors	A	12	10	25	20	0.011
10	I routinely address sexual health issues with all of my adolescent patients	S/K	39	31	54	43	0.014
11	*My sex/gender/sexual identity interferes with my ability to treat patients	A	107	86	94	75	0.015
12	*My patients do not want me to investigate sexual health problems	A	93	74	104	83	ns
13	Sexual health has an important impact on general and mental health	A	118	94	123	98	ns
14	*Talking about sexual health issues causes more trouble than it is worth	A	114	91	113	90	ns
15	*Regardless of what I say, patients will discontinue medications if they believe them to have sexual side effects	A	72	58	77	62	ns
16	*I am too pressed for time to routinely investigate sexual health issues	S/K	50	40	59	47	ns
17	*Patients with sexual concerns will spontaneously bring them up if they want me to know	A	92	74	93	74	ns
18	*Exploring sexual health issues with neuro-atypical and/or developmentally disabled patients causes me discomfort	A	44	35	51	41	ns
19	*I find it challenging to find developmentally appropriate information regarding sexual health for my pediatric patients and their parents or guardians	A	18	14	29	23	ns
20	*If I deal with the sexual health issues of minors, I will lose patients	A	110	88	111	89	ns

*A, attitudes; S/K, skills and knowledge*.

a Responses refer to number and percentage of participants who responded “Strongly agree” or “Agree” on a given item; for reverse-coded items (noted with an asterisk), participants responded “Strongly disagree,” or “Disagree.”

b *Calculated with the McNemar–test*.

The session was rated highly on a five-point Likert scale (4.6 +/− 0.7; Table 5. Finally, 116 participants (93%) provided responses to our optional question about the session in general, and about its video-based component in particular. Comments were generally positive, with representative verbatim quotes including the following: “We were very impressed by the quality of the talk, the videos were amazing for our modeling on how to approach sexual issues. I am very glad that we could take part in this class and hope we can keep participating in the future;” “This online multisite approach is a unique and effective contribution to training, particularly given the recent pandemic. Thank you for including our program;” and “The video interactions were particularly helpful in making the concepts ‘come alive' and providing actionable clinical examples to emulate and learn from: I will for sure be using your ‘electric/mechanical/plumbing' analogies [which refer to arousal/erection//ejaculation] in my own practice, and for that I thank you.” There were over a dozen requests to make the teaching materials available, including this representative one: “Videos were great, and it would be useful to have access to them along with a facilitator's guide [*a la* NNCI (National Neuroscience Curriculum Initiative)], so programs could develop their own seminars based off of this one.” There were several suggestions for future enhancements, most consistently the use of small break-out groups to increase interactivity. The use of synchronized videoconferencing had the lowest rating on the session evaluation, and the poor phrasing of our question may have introduced too high a threshold to overcome, as we inquired about “Zoom *contributing to* or *enhancing* the training in some way.” Despite this, ratings were still on average above 4 (“somewhat agree”), and textual comments were largely positive, noting more of its advantages, particularly the opportunity to interact with learners across institutions: “Loved having multiple sites; think this is great early move toward harnessing this technology.”

**Table 5:** Session evaluation (*n* = 125).

	**M**	**SD**
Video examples exemplifying/reinforcing the didactic content	4.7	0.6
Addressing its stated objectives	4.6	0.6
Overall usefulness/applicability/relevance?	4.6	0.6
Being interested in other didactic offerings using a similar approach	4.6	0.6
Likelihood to recommend this training to other peers	4.6	0.7
Overall educational content/“bang for your buck”	4.5	0.7
Zoom contributing or enhancing the training in some way	4.2	0.9
Summary rating	4.6	0.7

*Likert scale: Strongly agree, 5; Somewhat agree, 4; Neither agree nor disagree, 3; Somewhat disagree, 2; Strongly disagree, 1*.

## Discussion

This study was designed to examine: (1) a specialized educational resource on sexual health and its impact on learners' knowledge, skills, and attitudes; (2) the use of videotaped depictions of clinical interactions to complement the module's content; and (3) the deployment of educational content through a multisite initiative using synchronized videoconferencing. The results of our study support the utility of each of the three components.

### *Sex Ed 201*: An Educational Resource Tailored for Child and Adolescent Psychiatrists

In keeping with experiences reported in the medical education literature in medicine (9–13), including psychiatry (32, 33), the CAP participants in our study felt uneasy and insufficiently prepared to address issues of sexuality in their clinical practice. Although realizing how “important” and “necessary” the topic was, they almost universally felt “awkward” or “uncomfortable” in broaching it directly with patients and families. We found that a one-time education session of 90 min led to improvements in knowledge, skills and attitudes measurable after 2 weeks.

We developed the education module mindful of the specialty's specific needs, and in particular that of presenting information in an appropriate way to children and adolescents of different ages and developmental needs, and with their parents or guardians as clinically indicated. We found wide variability in clinicians' prior knowledge and education regarding sexual health, and inconsistency in the resources available to them. Indeed, we found particular improvement after the training on concrete knowledge about the availability of developmentally tailored materials, including books, websites, trainings, and others such as those we curated. This finding highlights the need to incorporate easily accessible and up to date resources into sexual health curricula to the extent possible. This is especially relevant for CAPs, who routinely interface with children, adolescents and adults with a broad array of age, language abilities, and developmental differences. We emphasized that addressing sexual health is important for all child and adolescent patients, no matter what diagnosis or (dis)ability they may have, but given time constraints, chose to provide more in-depth education on only one diagnosis. We decided on and incorporated autism-specific content given the growing (and overdue) attention to the importance of sexual health among youth and young adults on the autism spectrum (28, 34).

The CAP training competencies, as outlined by the American Board of Psychiatry and Neurology (35) and by the Accreditation Council for Graduate Medical Education's program requirements (36) include several aspects relevant to sexuality, including among others: psychosexual development, disorders of sexual development, sexual abuse, and sexual orientation. However, sexual function, sexual health, and common sexual dysfunctions tend to be sparely addressed, when addressed at all. There are no clear parameters on what a comprehensive curriculum would entail, or how it could be incorporated into an already overflowing set of training requirements. Moreover, there is a dearth of CAP educators appropriately trained in sexual health. To that end, our initiative provides a concrete step toward greater standardization in education and training (37), as well as material that can be incorporated and adapted “off the shelf” by end users who may otherwise not have ready access to content experts.

### *Making It Real*: Incorporating Simulation With Standardized Patients Into CAP

Simulation with SPs offers learners an opportunity to gain exposure to a wide range of patients and clinical scenarios, and to practice and refine clinical skills in a safe and supportive environment (38). Although widely used in undergraduate medical education, simulation-based training in post-graduate psychiatric training is considerably more limited (39–41). SP use in CAP training is virtually non-existent, partly because of the additional practical, legal, and ethical considerations inherent to working with underage actors (42). Still, there are several informative examples of simulation-based psychiatric training using SPs in other fields. For example, performance on adolescent suicide risk assessment among residents in pediatrics objectively improved after using an SP-based learning module (43).

By incorporating videotaped depictions of experienced clinicians interacting with SPs, we were able to offer realistic portrayals of encounters likely to be faced by CAPs in their clinical practice. Learners were able to see the techniques and actual words used to demystify sexual health and turn it into a routine part of outpatient visits. In an ideal setting, learners would be able to practice these skills during face-to-face interactions with SPs. However, such an approach would be logistically taxing, particularly for as many participants and given the challenges to provide SPs around as many sites with varying levels of local resources and expertise. Instead, the availability of enduring learning materials such as ours allows for a series of consistent and standardized stimuli, which can then be adapted locally and incorporated into practice by individual learners or training programs.

We consider that the availability of such resources, especially if part of broader materials, could strengthen learning opportunities across the discipline. For example, curated content incorporating SP depictions could be useful to ensure consistent training across training sites, regardless of their size or local resources. Aspects of clinical practice with high public health impact and limited local expertise (such as adolescent substance abuse) could be natural topics to consider as next steps. The challenges to incorporate underage SPs notwithstanding, we are committed to expanding simulation in CAP. We are not alone in seeing the potential that could be unleashed: “The ability of mental health simulation to bridge the gap between education and clinical practice, alongside its potential for interprofessional education and initial evidence supporting its effectiveness, merit its inclusion as a key educational tool in providing better care for mental health needs…Mental health simulation is poised to have a positive effect should the necessary support, funding, and progressive thinking be applied” (44).

### *The Zoom Where It Happens*: Leveraging a New Technology to Enhance CAP Training

The timing of our scheduled training session vis-á-vis the COVID-19 pandemic forced us to move from what had initially been designed as a single site initiative to one that incorporated 15 peer institutions. Were it not for the pandemic, it is unlikely that we would have conceptualized this effort as one delivered through synchronous videoconferencing. The approach provided several unique advantages: a broader reach in the number and geographic extraction of participants; training consistency across sites; interaction in real time with content experts and fellow learners; and logistic ease in securing curricular content and experienced faculty. Our resort to this means of content delivery is not new (45), and we are in good company with other medical specialties that adapted rapidly to the limitations brought on by the pandemic [e.g., (46)].

Apart from those aspects of sexual health we set out to study, we consider this report a proof of principle for a novel education model in CAP. The combination of a secure virtual platform for synchronous videoconferencing, and the recruitment reach available through a network such as AALI, together offer entirely new opportunities. Given the relatively small size of the field, and the limited number of faculty experienced in a range of subspecialized topics, we believe that CAP could stand to benefit from similar efforts focused on strategic content areas.

### Limitations

We recognize several limitations to our study. First, our sample was modest in size and varied in its characteristics, which may limit its representativeness and generalizability to other training programs within, and particularly outside of the US; second, we recognize the possibility of a social desirability bias: learners may have answered the surveys more favorably at the second time point; third, we did not have a control group, such as of learners exposed to the lecture content but not to the videos, which could have helped identify active components of the intervention; fourth, we recognize that even as we measured changes in attitudes and knowledge 2 weeks after the intervention, we do not know whether effects will prove to be durable, or whether they will translate into actual clinical practice. A single exposure may not be sufficient to have an enduring effect; a spiral curriculum could be beneficial, wherein repeat encounters with sexual health content as relevant to CAP would reinforce previous learning. Compounding these measurement limitations is the fact that our primary outcome measure is a practical adaptation that does not have the strong empirical validation and support we would have optimally worked with. Finally, in harnessing Zoom at the early stages of the pandemic, we had much to learn. As aptly noted by one of the participants: “We have miles to go before we sleep regarding taking optimal advantage of this approach, which we absolutely should.” In particular, future iterations of this approach should carefully balance how best to incorporate interactive components, including optimal use of break-out rooms.

In summary, our study shows that a sexual health curriculum enriched by video-based examples can lead to measurable improvement in outcomes pertinent to the clinical practice of CAP. These educational materials are available for distribution, use and adaptation by local instructors. Our study also provides proof-of-principle for the use of multisite educational initiatives through synchronized videoconferencing.

## Data Availability Statement

The raw data supporting the conclusions of this article will be made available by the authors, without undue reservation.

## Ethics Statement

This study was approved by The Yale Human Investigations Committee (Protocol # 2000027918). It was deemed exempt, with survey completion standing for tacit consent. Written informed consent was not required for this study, in accordance with national legislation and institutional requirements.

## Author Contributions

LD and EG designed the sexual health curriculum and wrote the scripts. LD, EG, and AM worked with standardized patients in developing the videotaped depictions, designed the study, and drafted the first version of the manuscript. AM took the lead in analyzing the data and is responsible for the integrity of the data and analyses. All authors recruited learners at their respective training sites and participated in the education module, reviewed and contributed to working drafts, and approved the final, submitted version.

## Conflict of Interest

The authors declare that the research was conducted in the absence of any commercial or financial relationships that could be construed as a potential conflict of interest.
