# Supplementary material for: Sexual Health in Child and Adolescent Psychiatry: Multi-Site Implementation Through Synchronized Videoconferencing of an Educational Resource Using Standardized Patients
Source: Front Psychiatry. 2020 Nov 17;11:593101. doi: 10.3389/fpsyt.2020.593101 (PMC7716796; doi:10.3389/fpsyt.2020.593101)
Supplement: Supplementary file 6 [file Presentation_1.PPTX]

## Slide 1
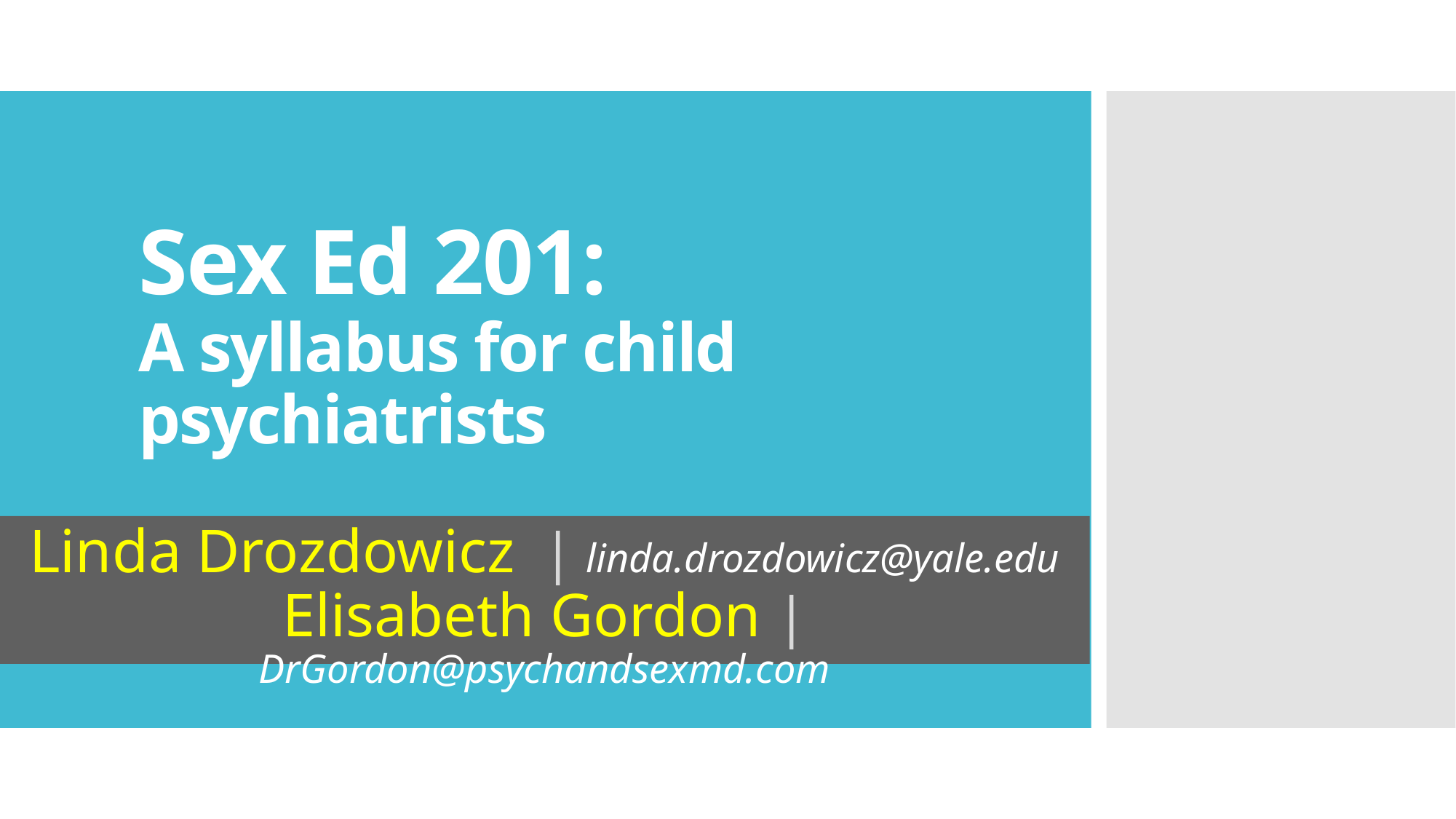

# Sex Ed 201: A syllabus for child psychiatrists
Linda Drozdowicz | linda.drozdowicz@yale.edu Elisabeth Gordon | DrGordon@psychandsexmd.com

## Slide 2
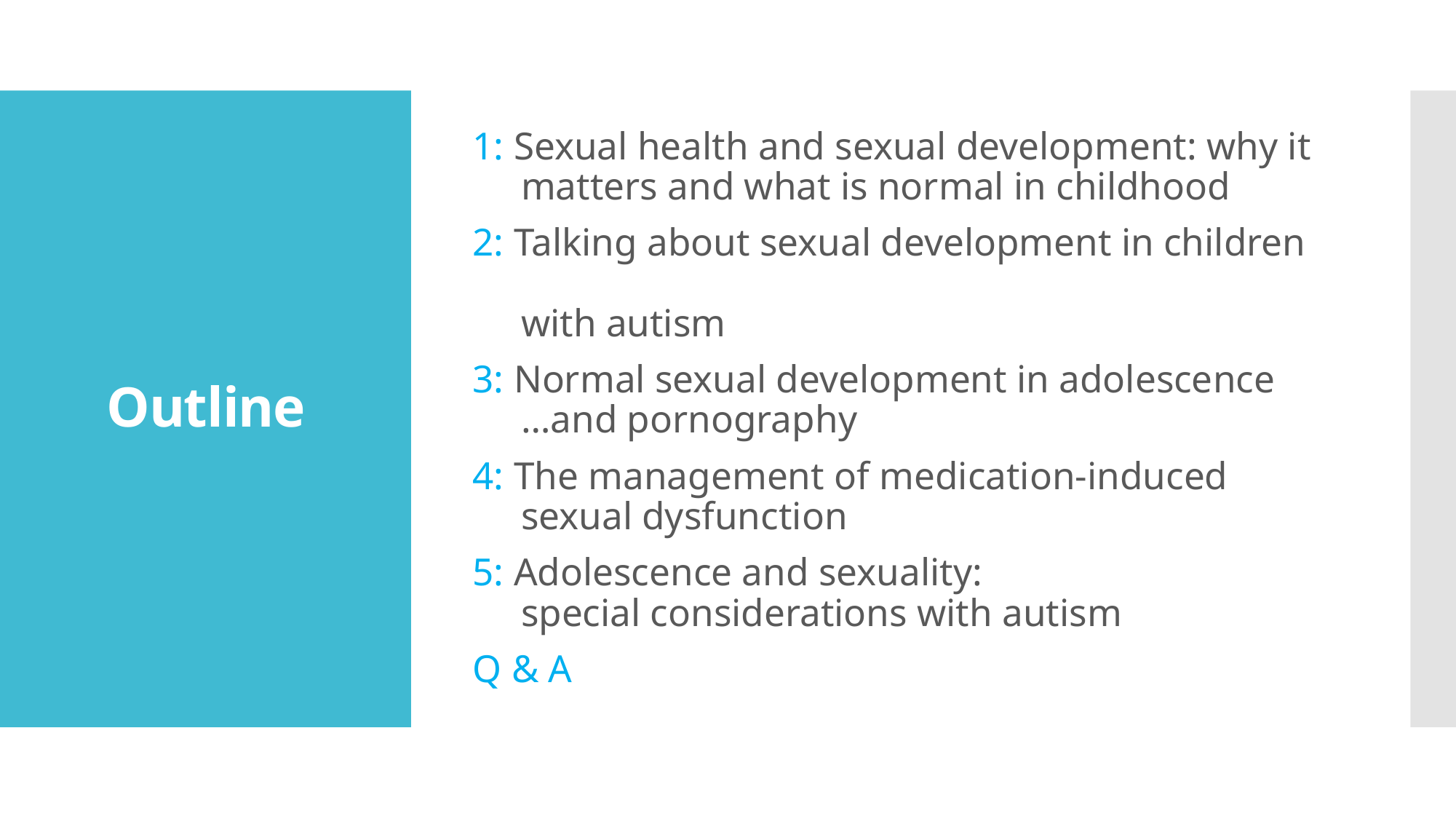

1: Sexual health and sexual development: why it
 matters and what is normal in childhood
2: Talking about sexual development in children
 with autism
3: Normal sexual development in adolescence
 …and pornography
4: The management of medication-induced
 sexual dysfunction
5: Adolescence and sexuality:
 special considerations with autism
Q & A
# Outline

## Slide 3
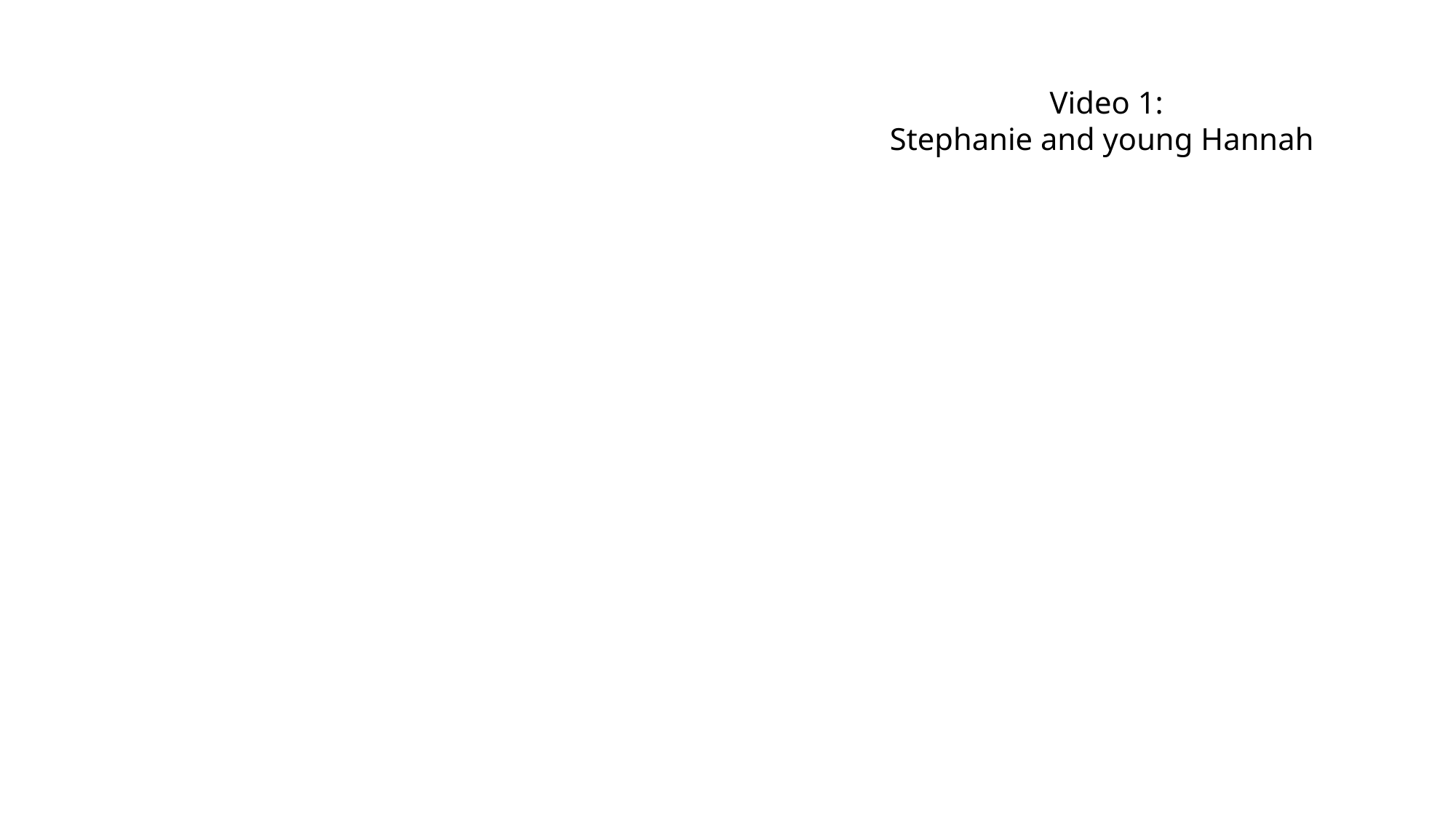

Video 1:
Stephanie and young Hannah 1

## Slide 4
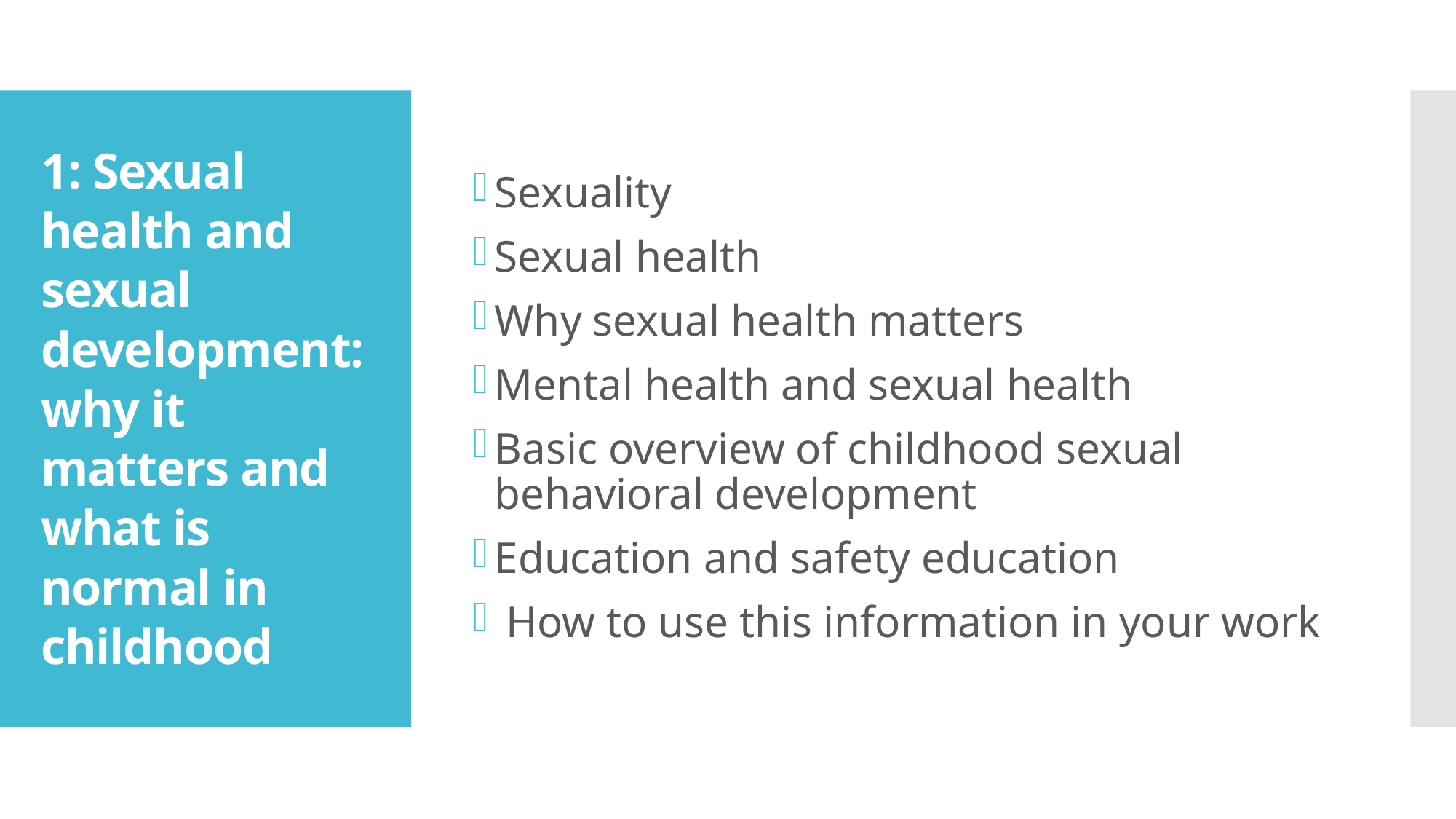

Sexuality
Sexual health
Why sexual health matters
Mental health and sexual health
Basic overview of childhood sexual behavioral development
Education and safety education
 How to use this information in your work
# 1: Sexual health and sexual development: why it matters and what is normal in childhood

## Slide 5
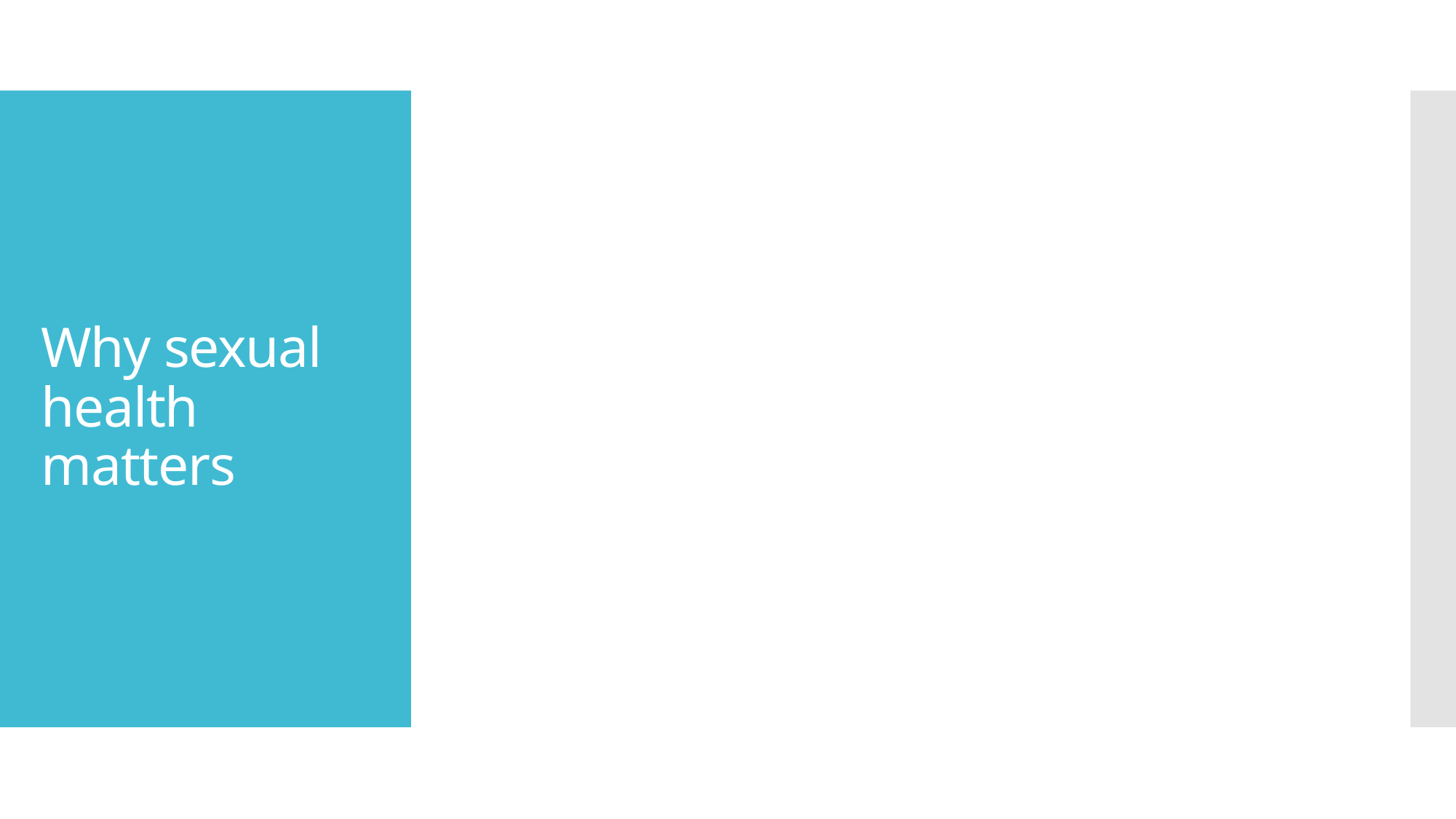

# Why sexual health matters

## Slide 6
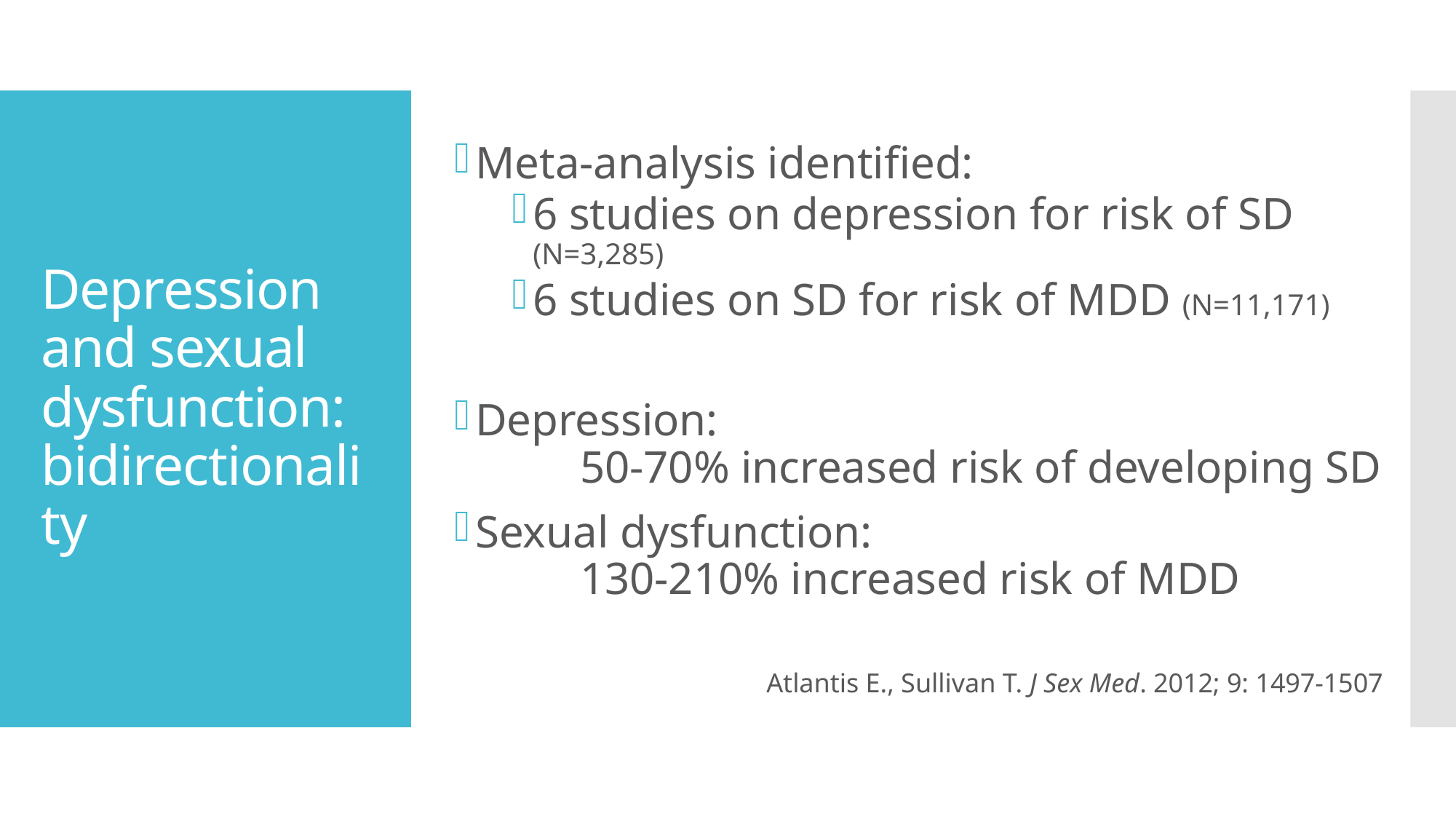

Meta-analysis identified:
6 studies on depression for risk of SD (N=3,285)
6 studies on SD for risk of MDD (N=11,171)
Depression:
	50-70% increased risk of developing SD
Sexual dysfunction:
	130-210% increased risk of MDD
Atlantis E., Sullivan T. J Sex Med. 2012; 9: 1497-1507
# Depression and sexual dysfunction: bidirectionality

## Slide 7
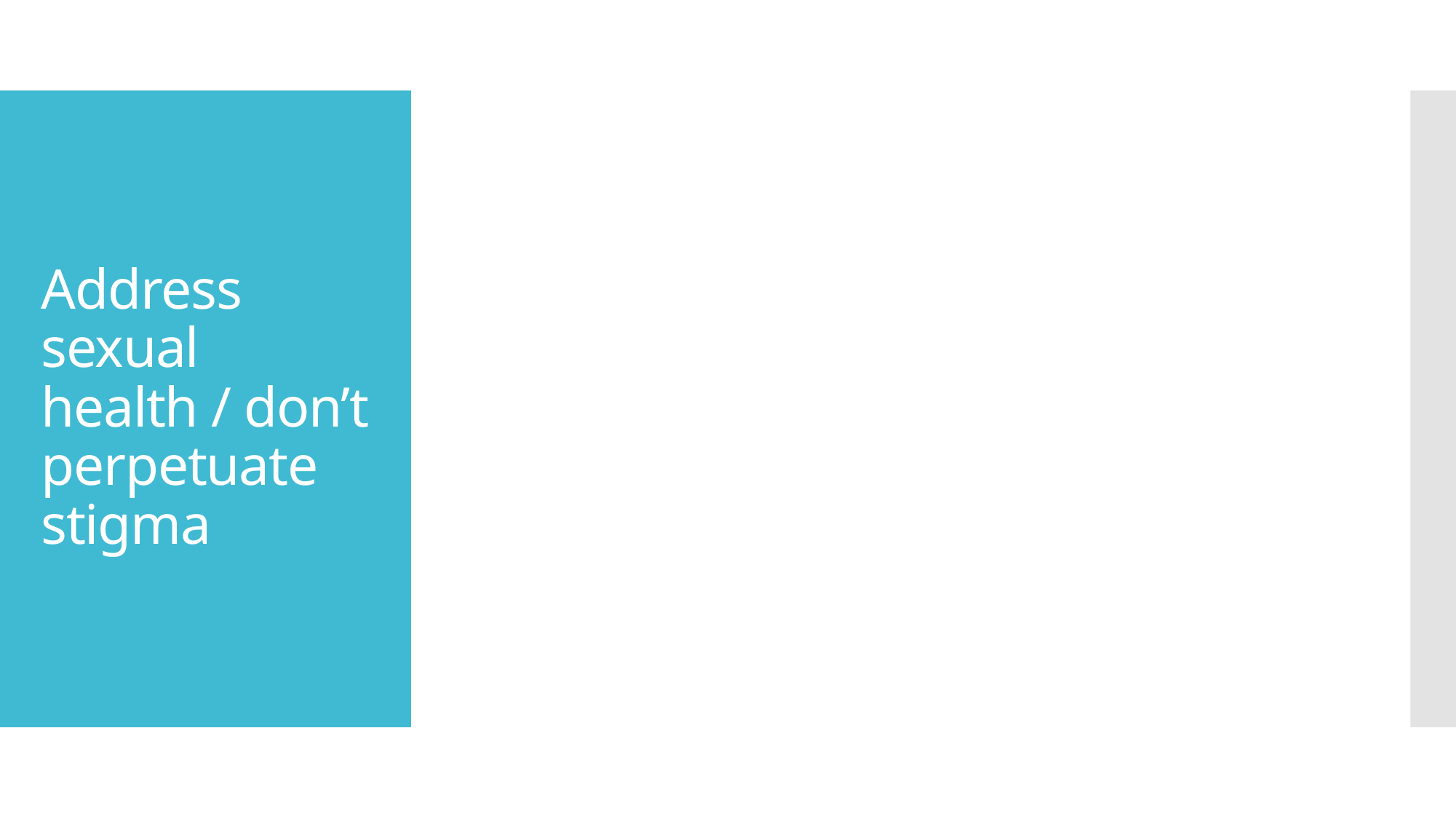

# Address sexual health / don’t perpetuate stigma

## Slide 8
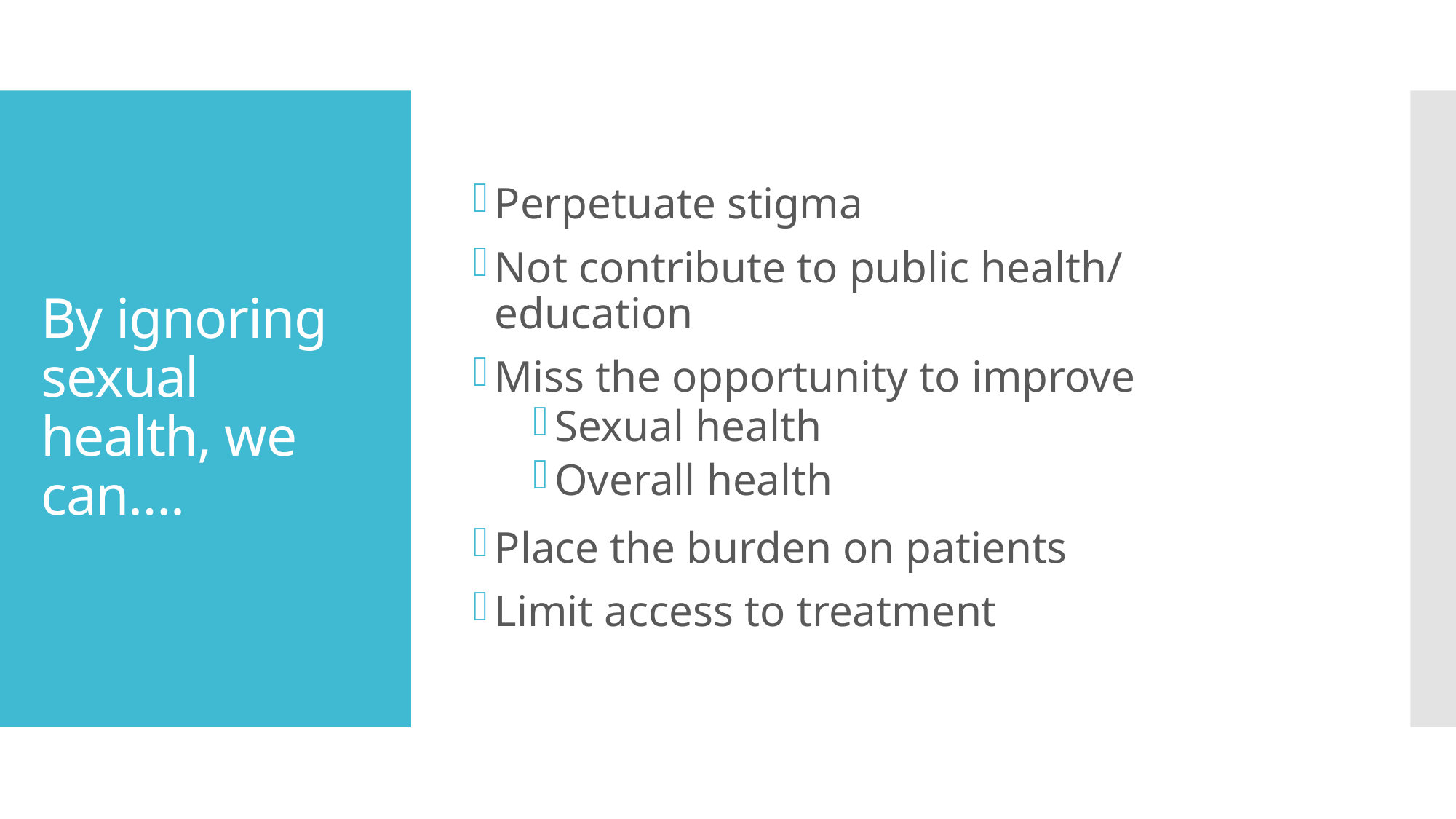

Perpetuate stigma
Not contribute to public health/ education
Miss the opportunity to improve
Sexual health
Overall health
Place the burden on patients
Limit access to treatment
# By ignoring sexual health, we can….

## Slide 9
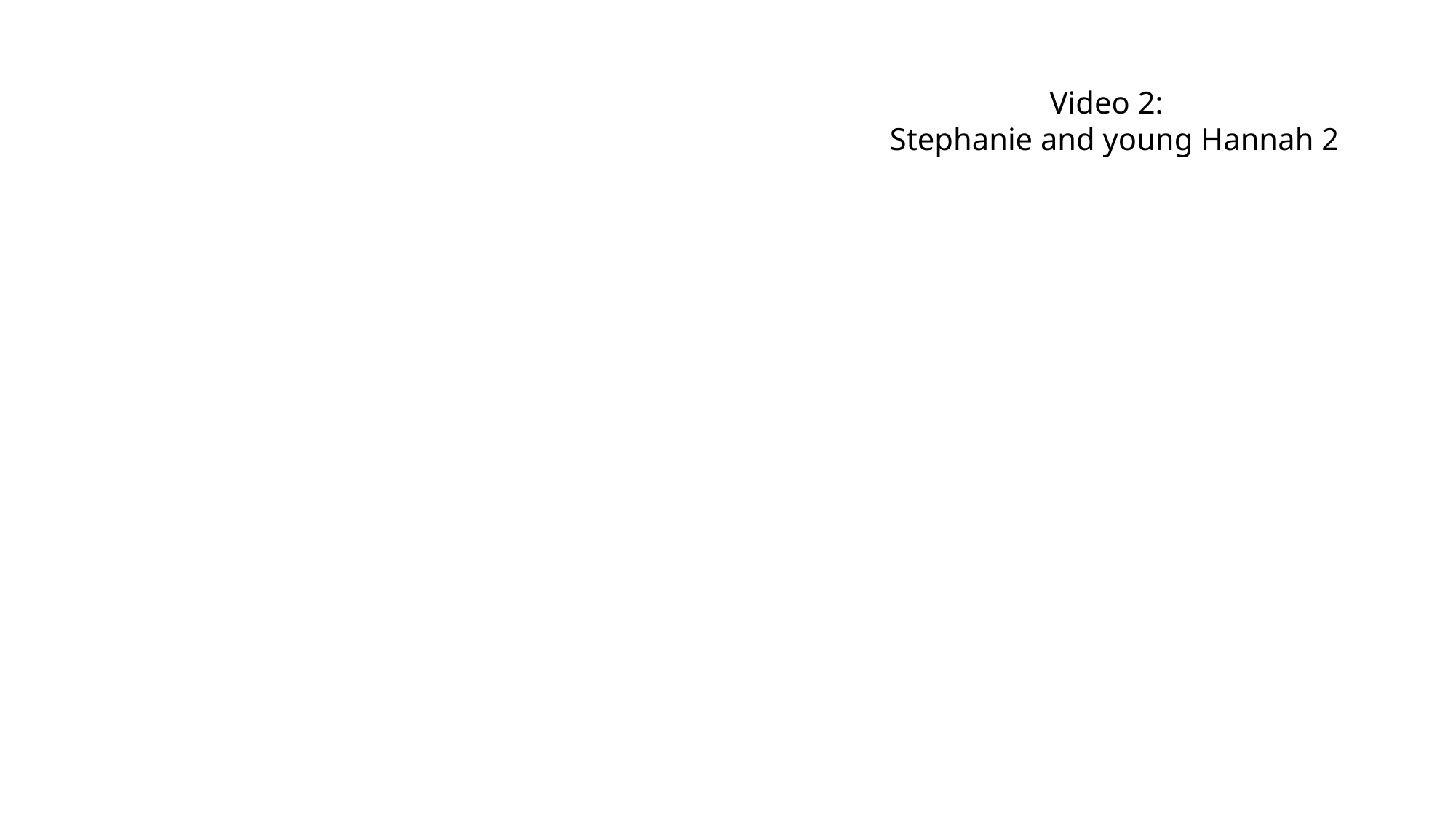

Video 2:
Stephanie and young Hannah 2

## Slide 10
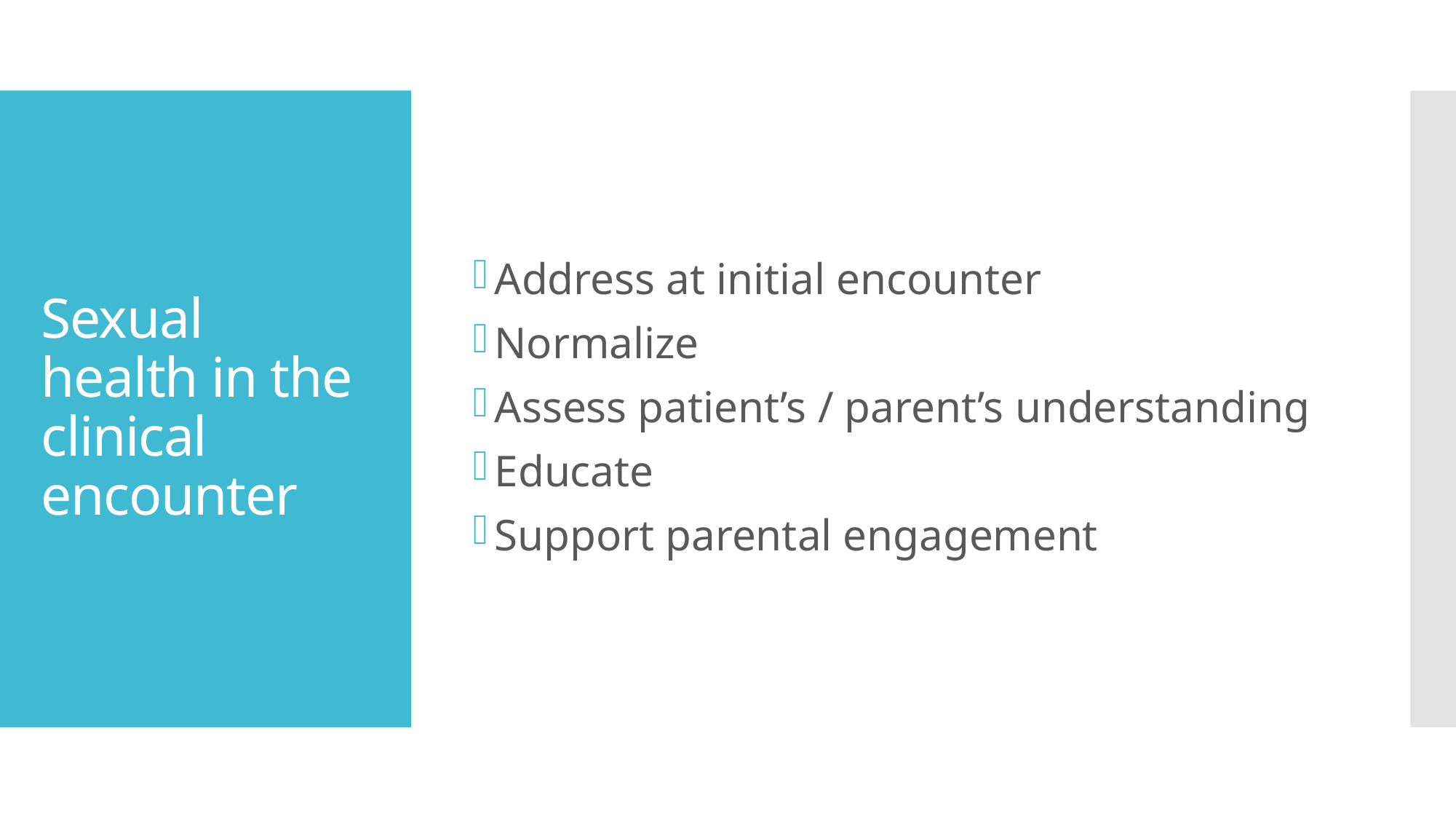

Address at initial encounter
Normalize
Assess patient’s / parent’s understanding
Educate
Support parental engagement
# Sexual health in the clinical encounter

## Slide 11
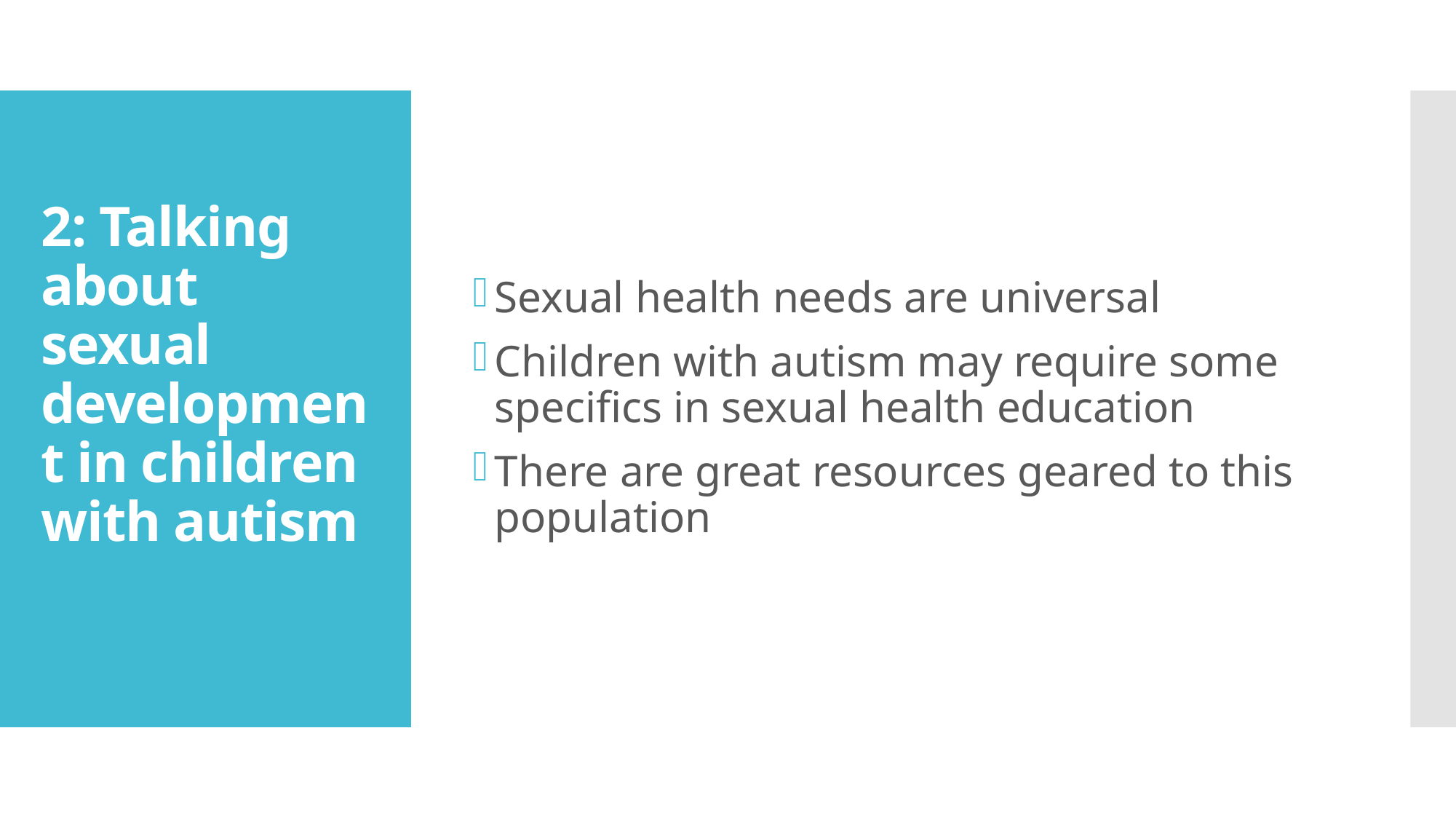

Sexual health needs are universal
Children with autism may require some specifics in sexual health education
There are great resources geared to this population
# 2: Talking about sexual development in children with autism

## Slide 12
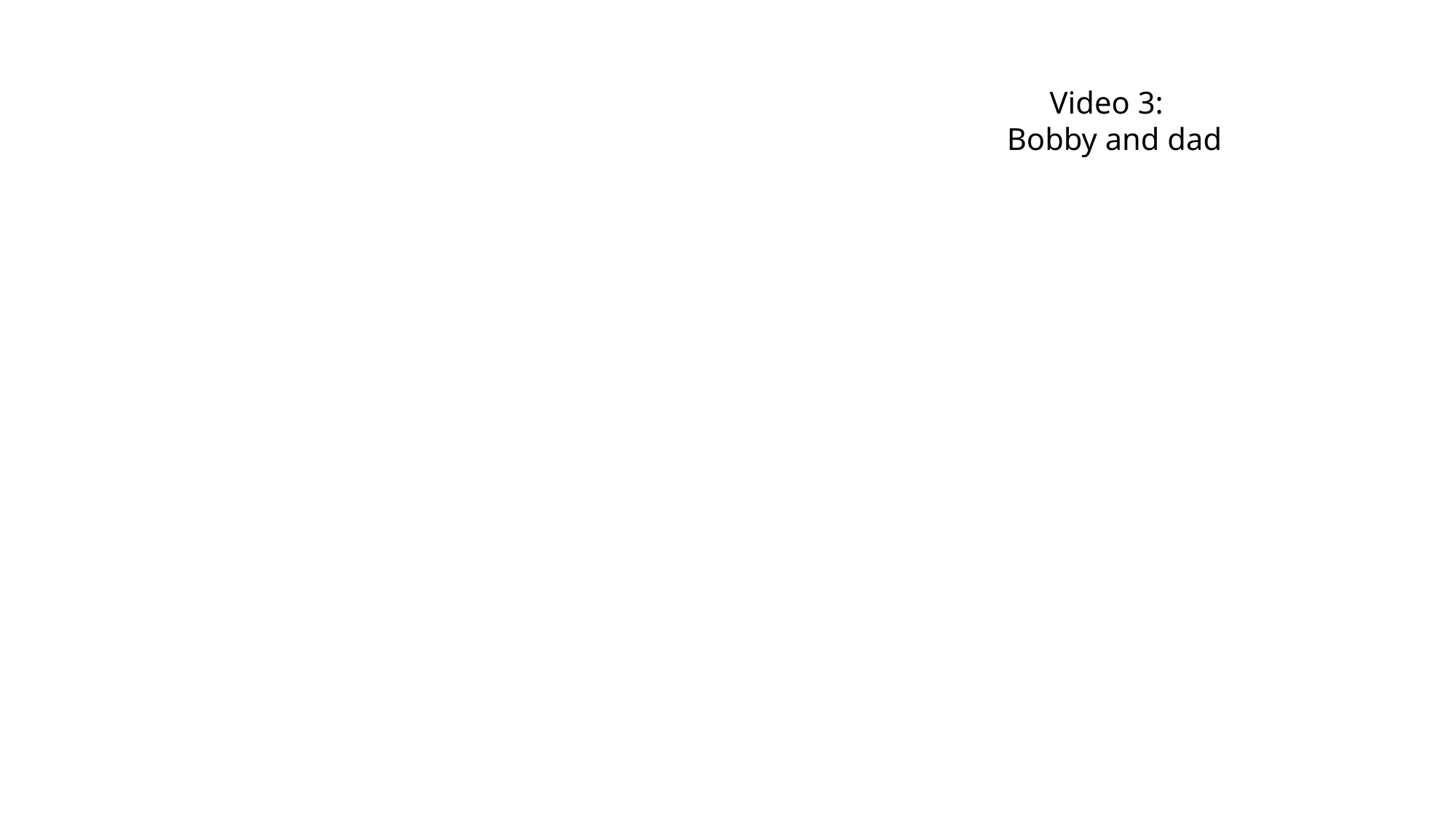

Video 3:
Bobby and dad

## Slide 13
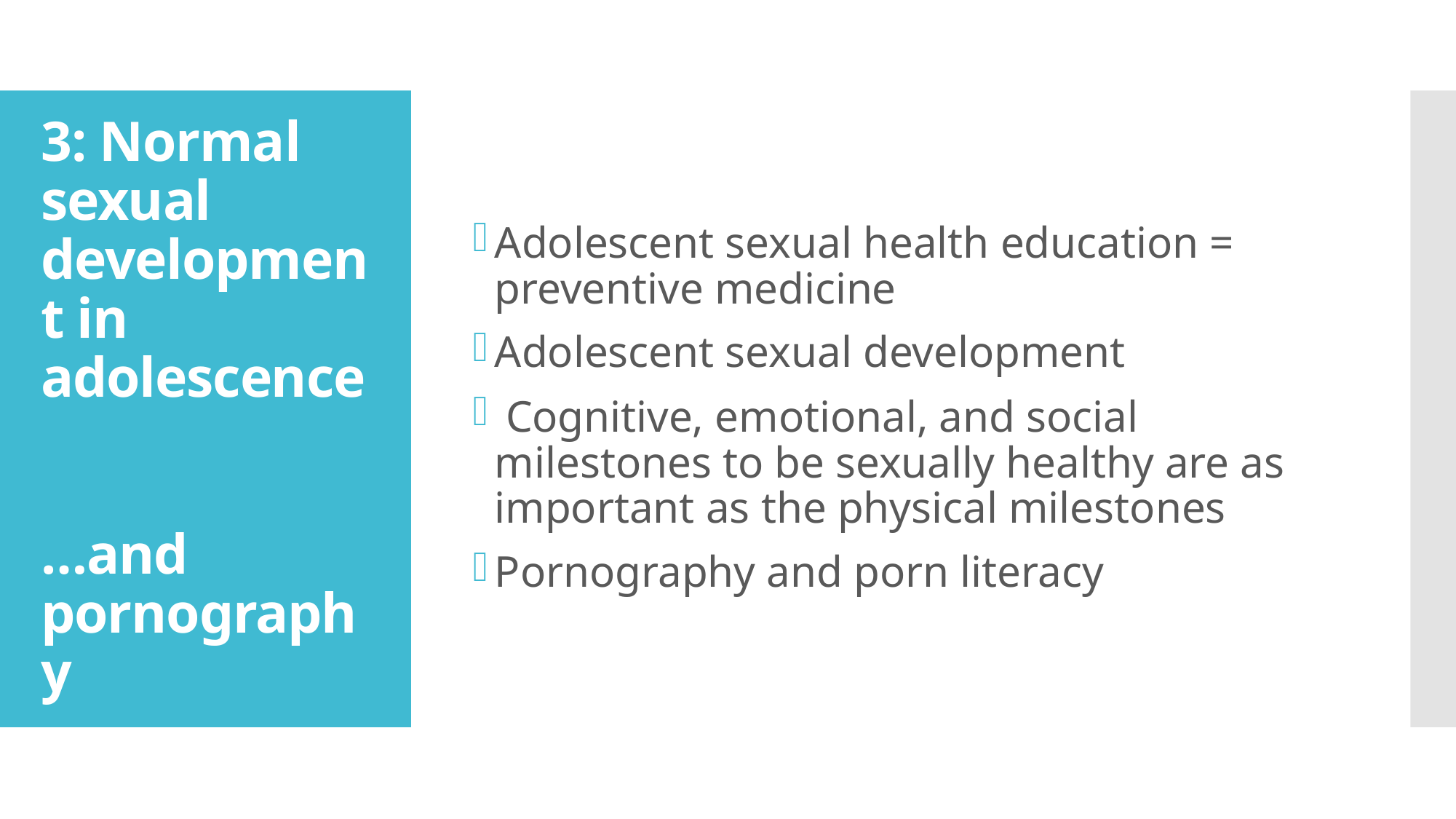

Adolescent sexual health education = preventive medicine
Adolescent sexual development
 Cognitive, emotional, and social milestones to be sexually healthy are as important as the physical milestones
Pornography and porn literacy
# 3: Normal sexual development in adolescence …and pornography

## Slide 14
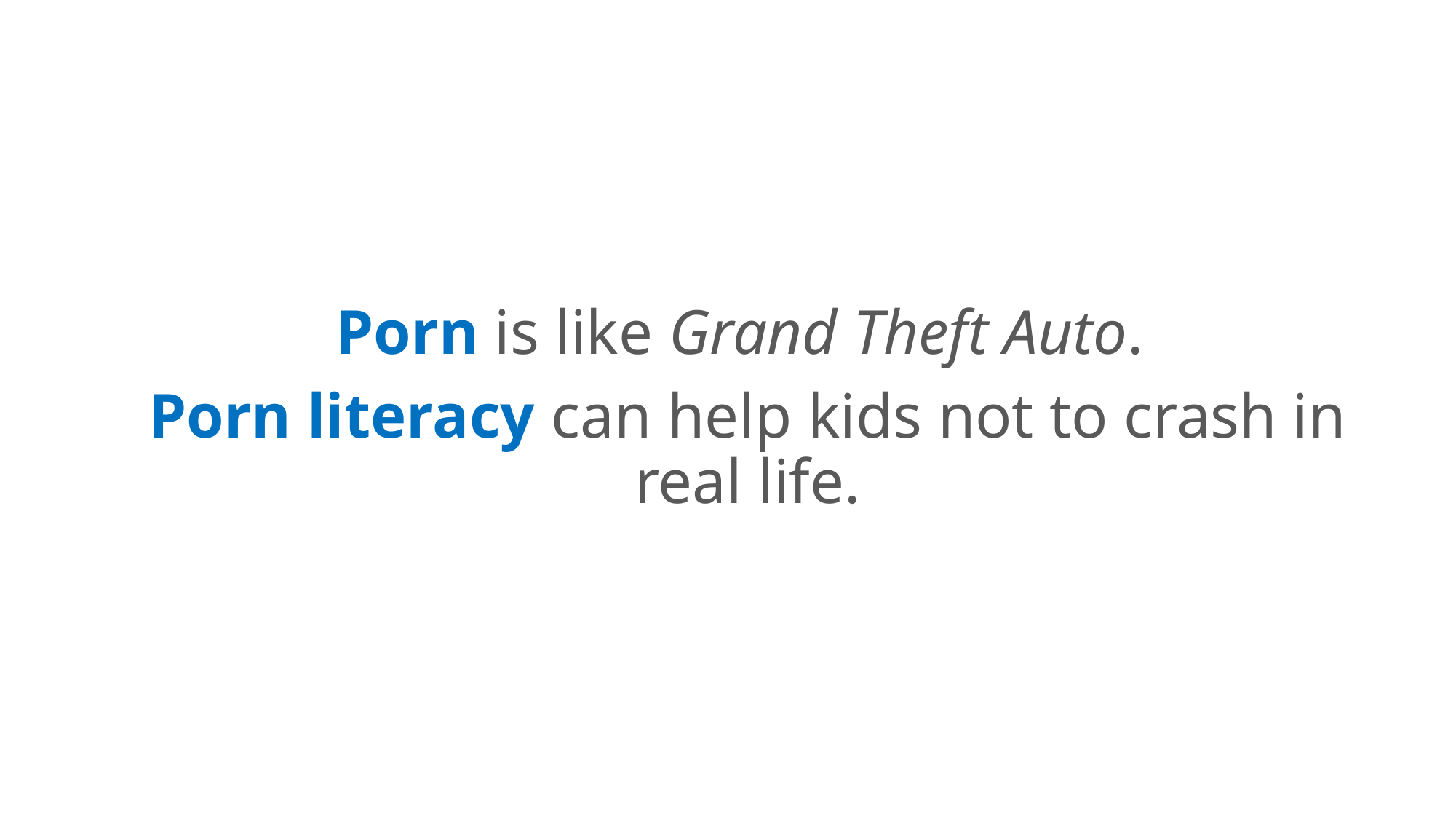

Porn is like Grand Theft Auto.
Porn literacy can help kids not to crash in real life.

## Slide 15
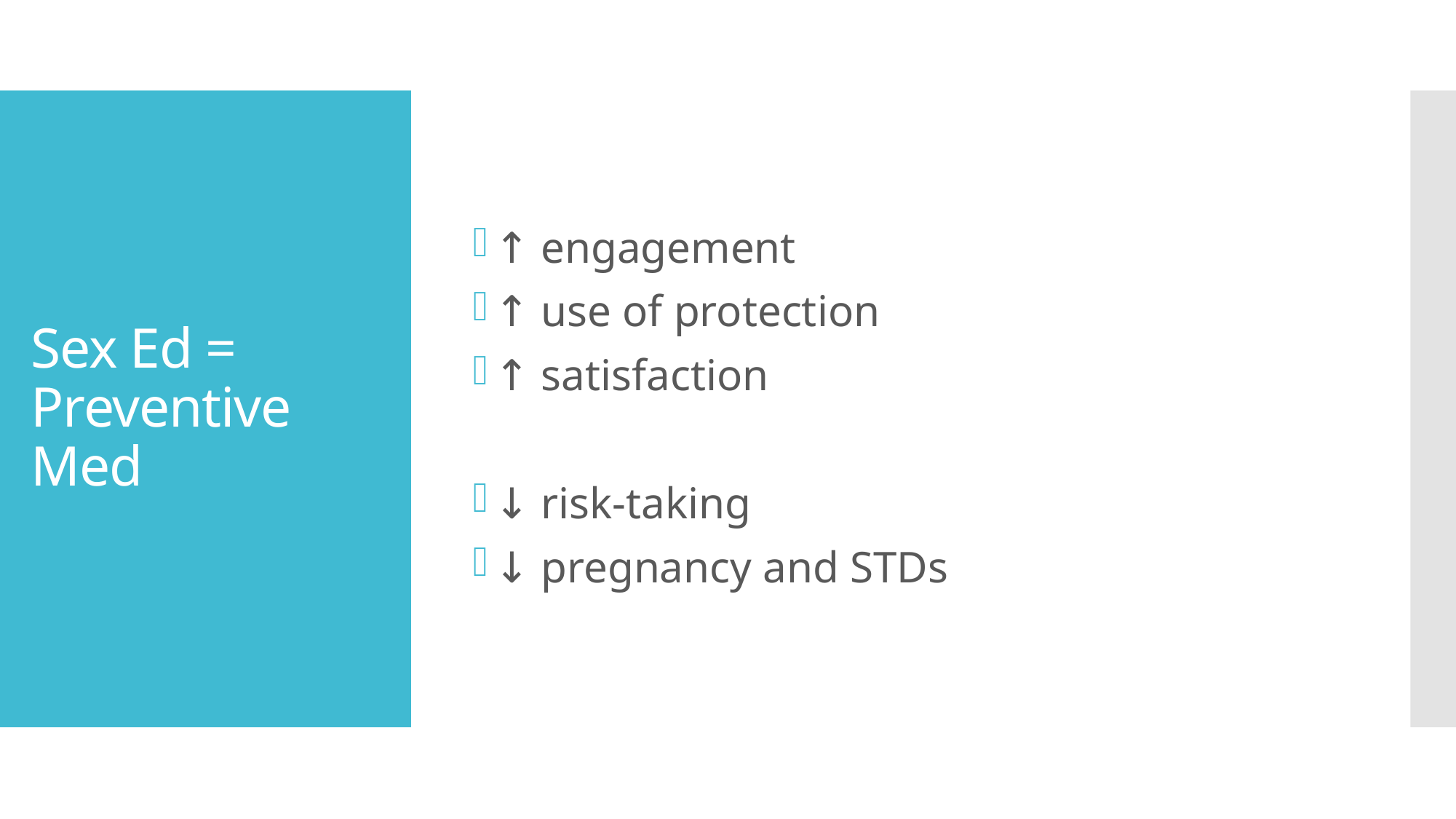

↑ engagement
↑ use of protection
↑ satisfaction
↓ risk-taking
↓ pregnancy and STDs
# Sex Ed = Preventive Med

## Slide 16
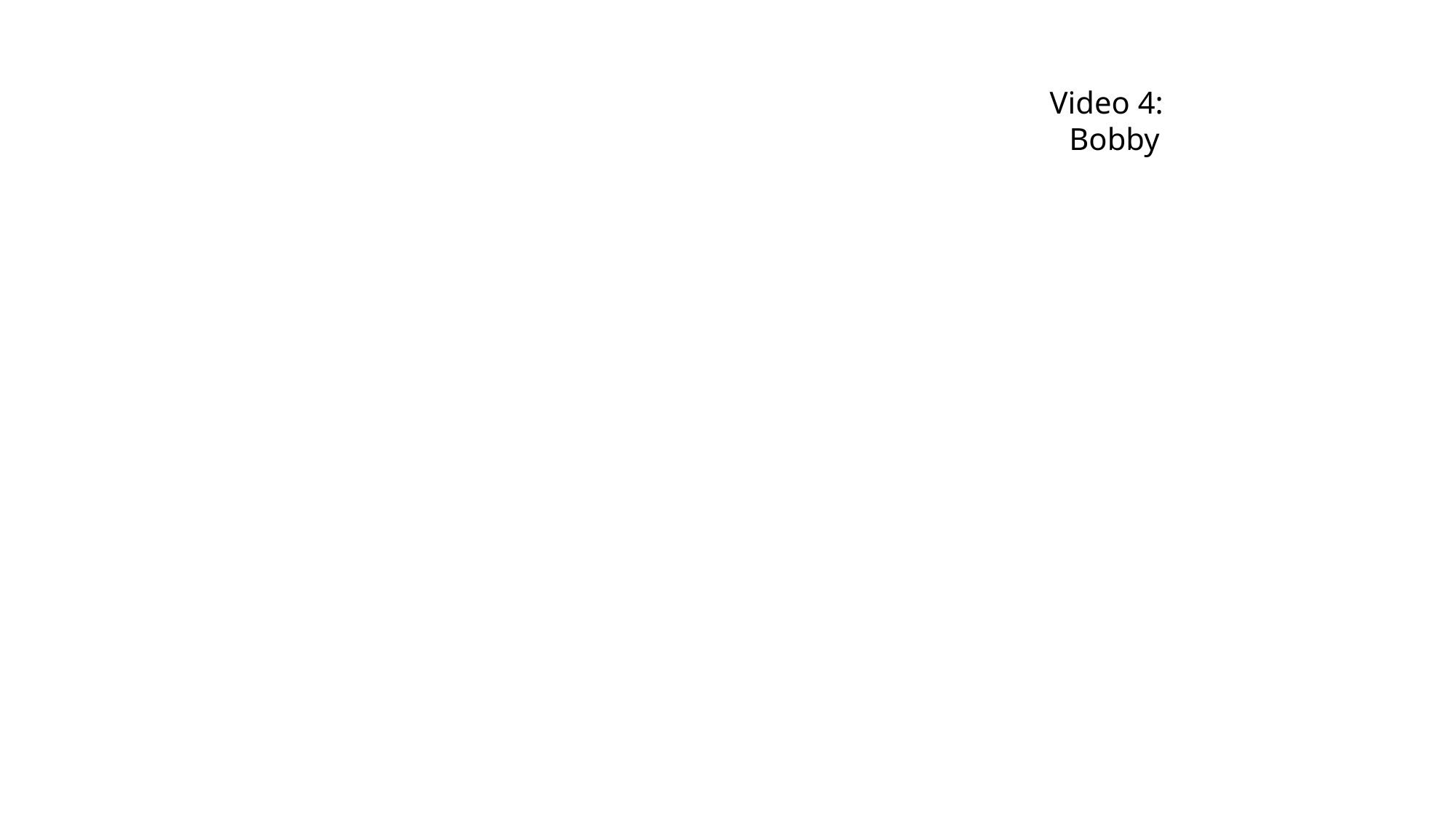

Video 4:
Bobby

## Slide 17
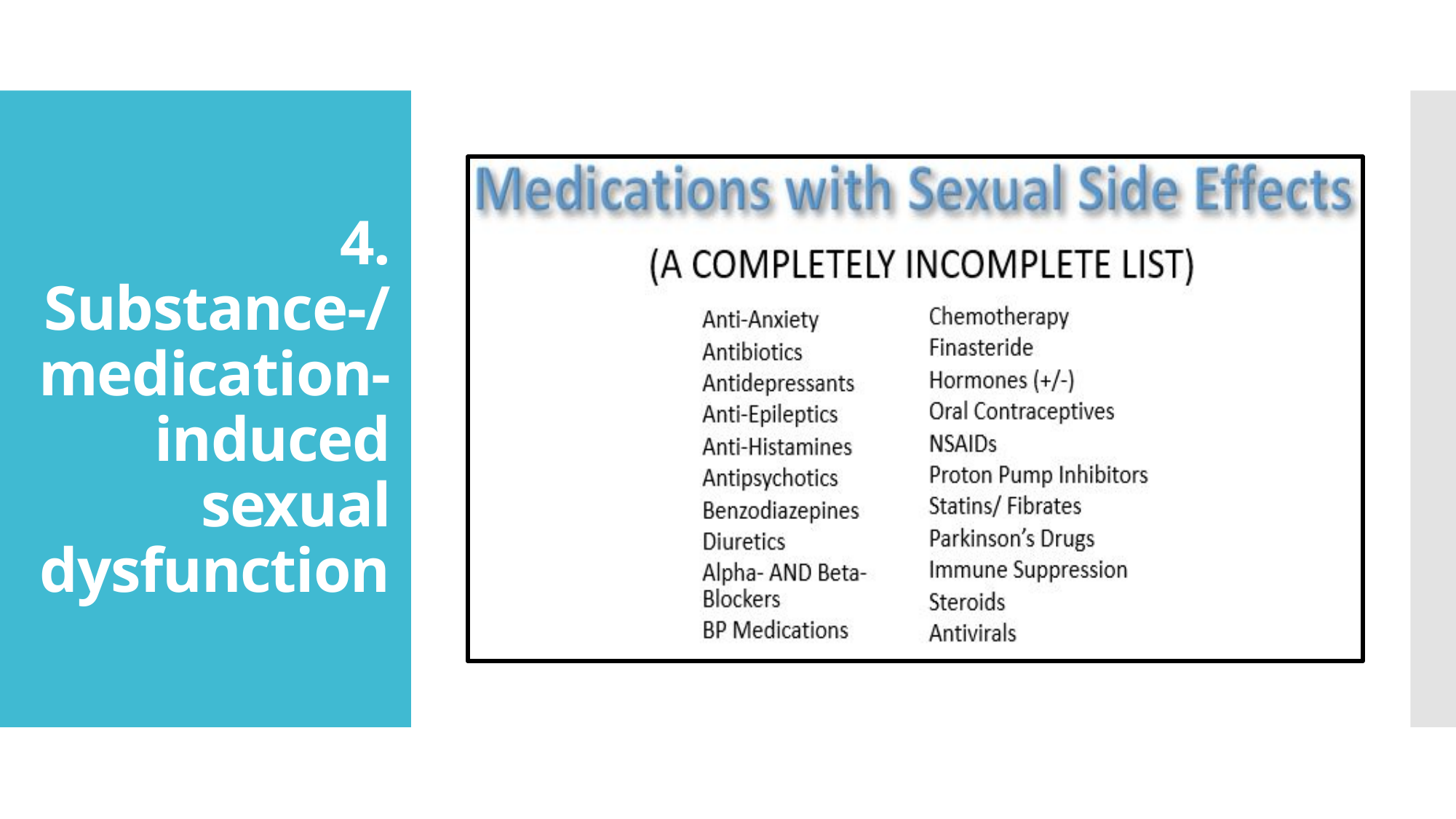

# 4. Substance-/ medication-induced sexual dysfunction

## Slide 18
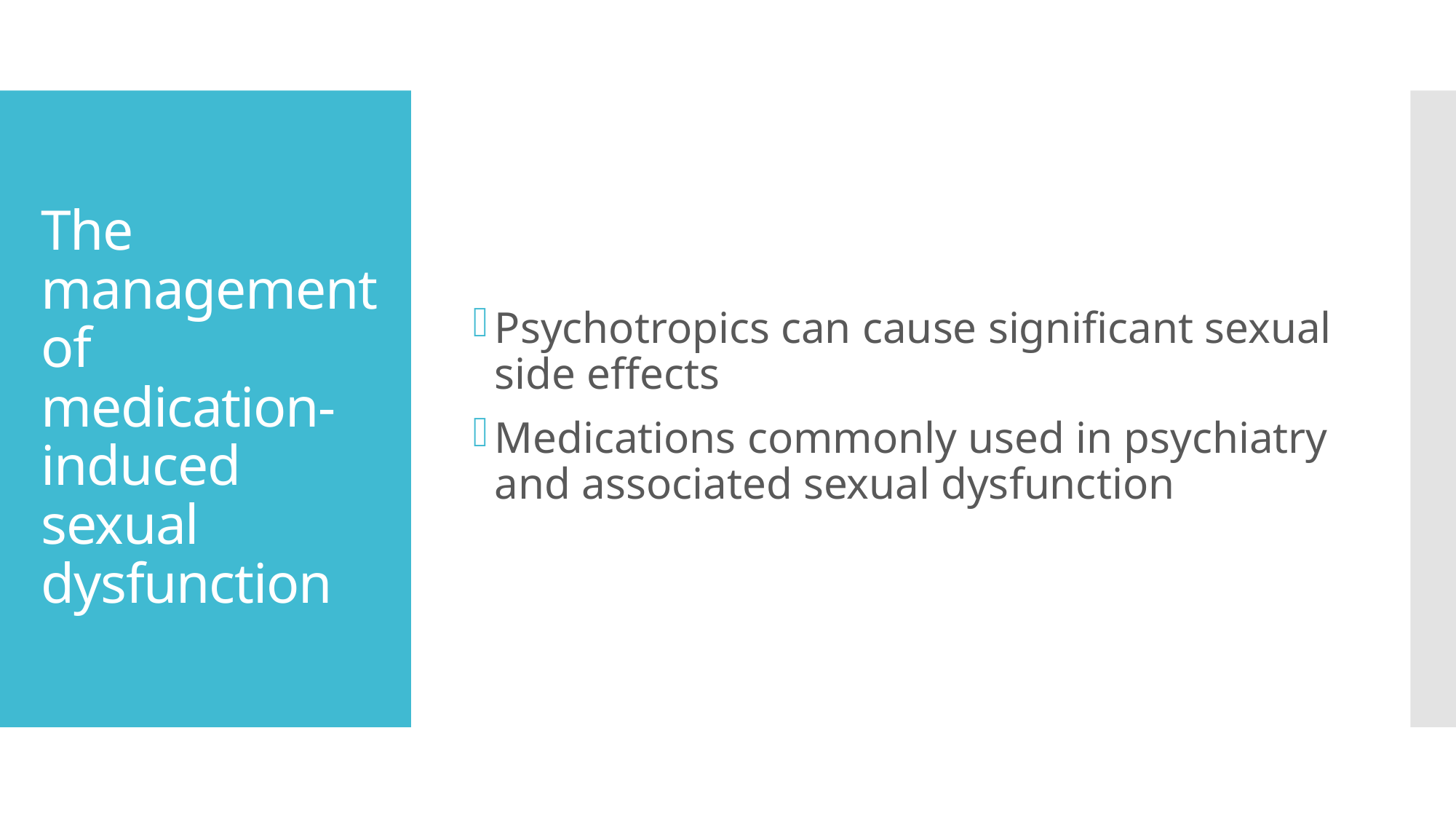

Psychotropics can cause significant sexual side effects
Medications commonly used in psychiatry and associated sexual dysfunction
# The management of medication- induced sexual dysfunction

## Slide 19
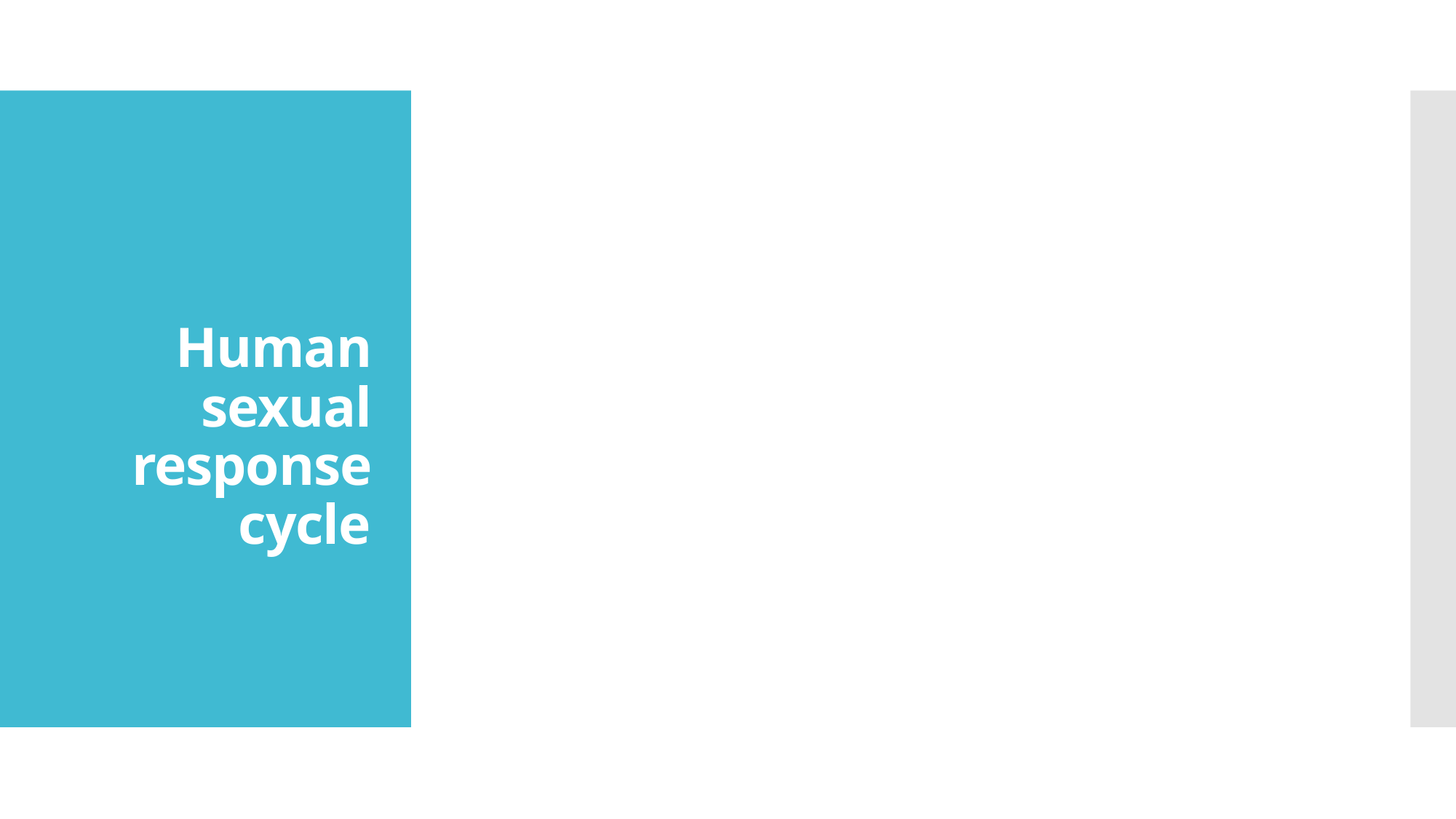

# Human sexual response cycle

## Slide 20
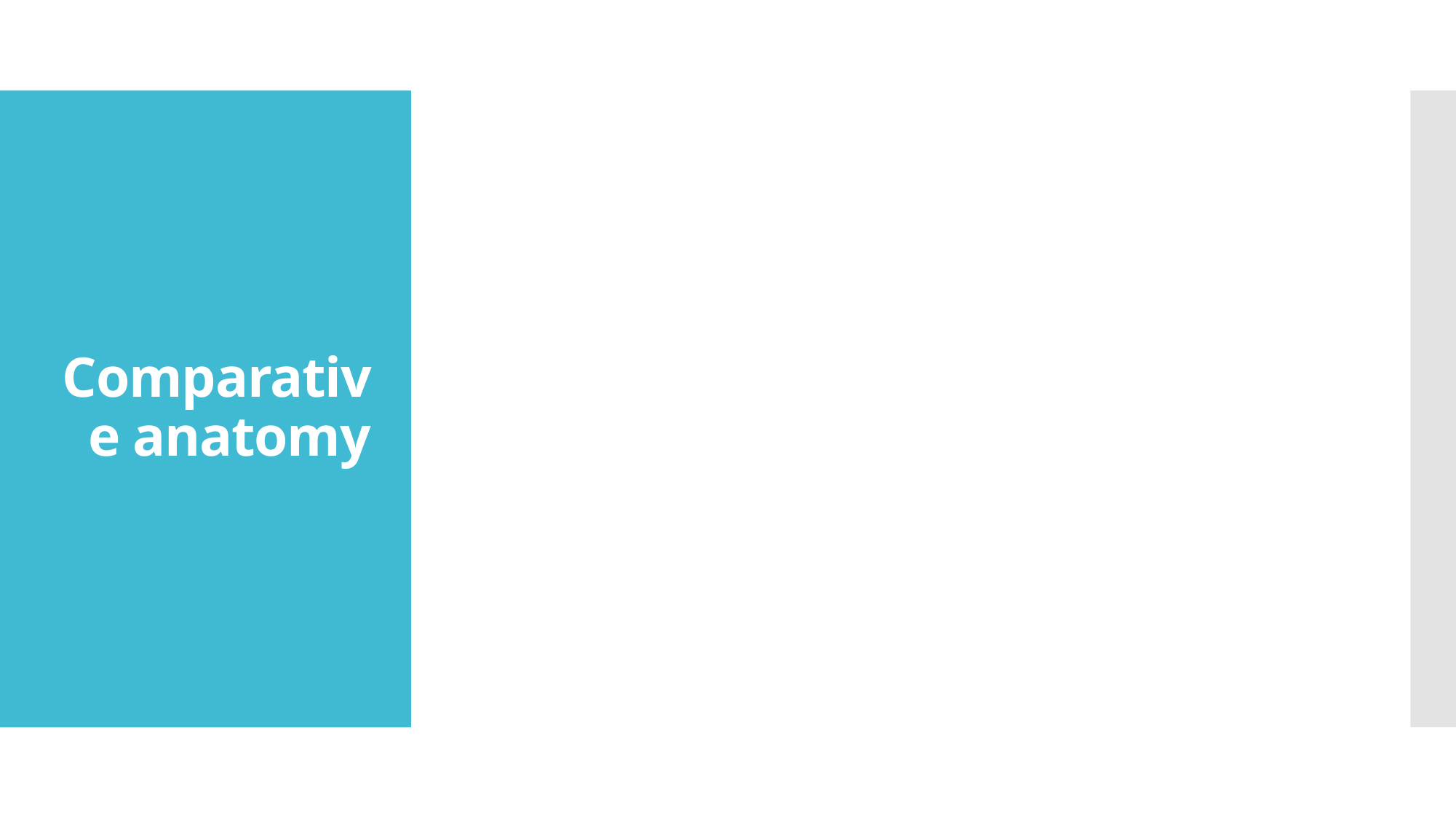

# Comparative anatomy

## Slide 21
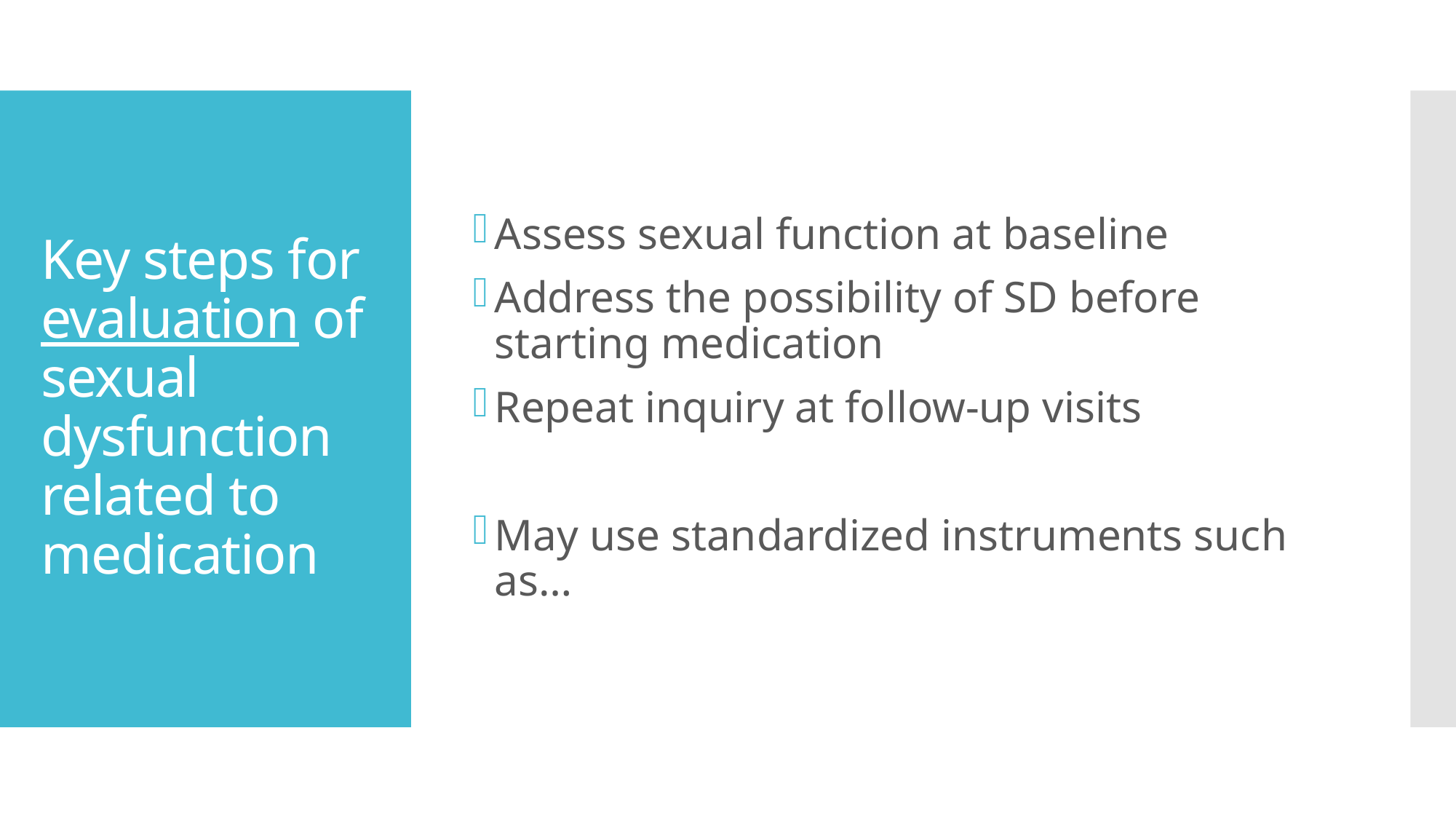

Assess sexual function at baseline
Address the possibility of SD before starting medication
Repeat inquiry at follow-up visits
May use standardized instruments such as…
# Key steps for evaluation of sexual dysfunction related to medication

## Slide 22
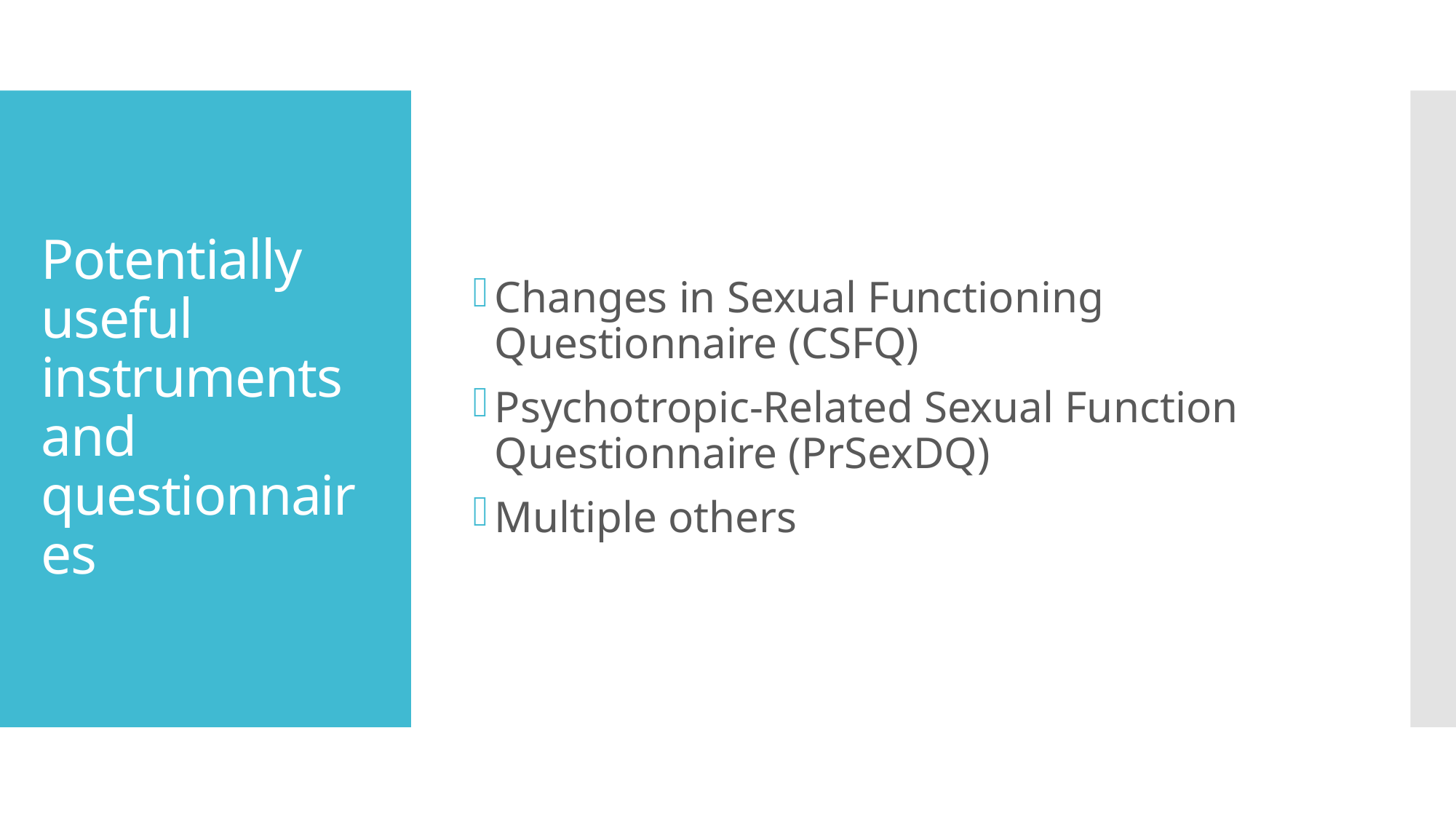

Changes in Sexual Functioning Questionnaire (CSFQ)
Psychotropic-Related Sexual Function Questionnaire (PrSexDQ)
Multiple others
# Potentially useful instruments and questionnaires

## Slide 23
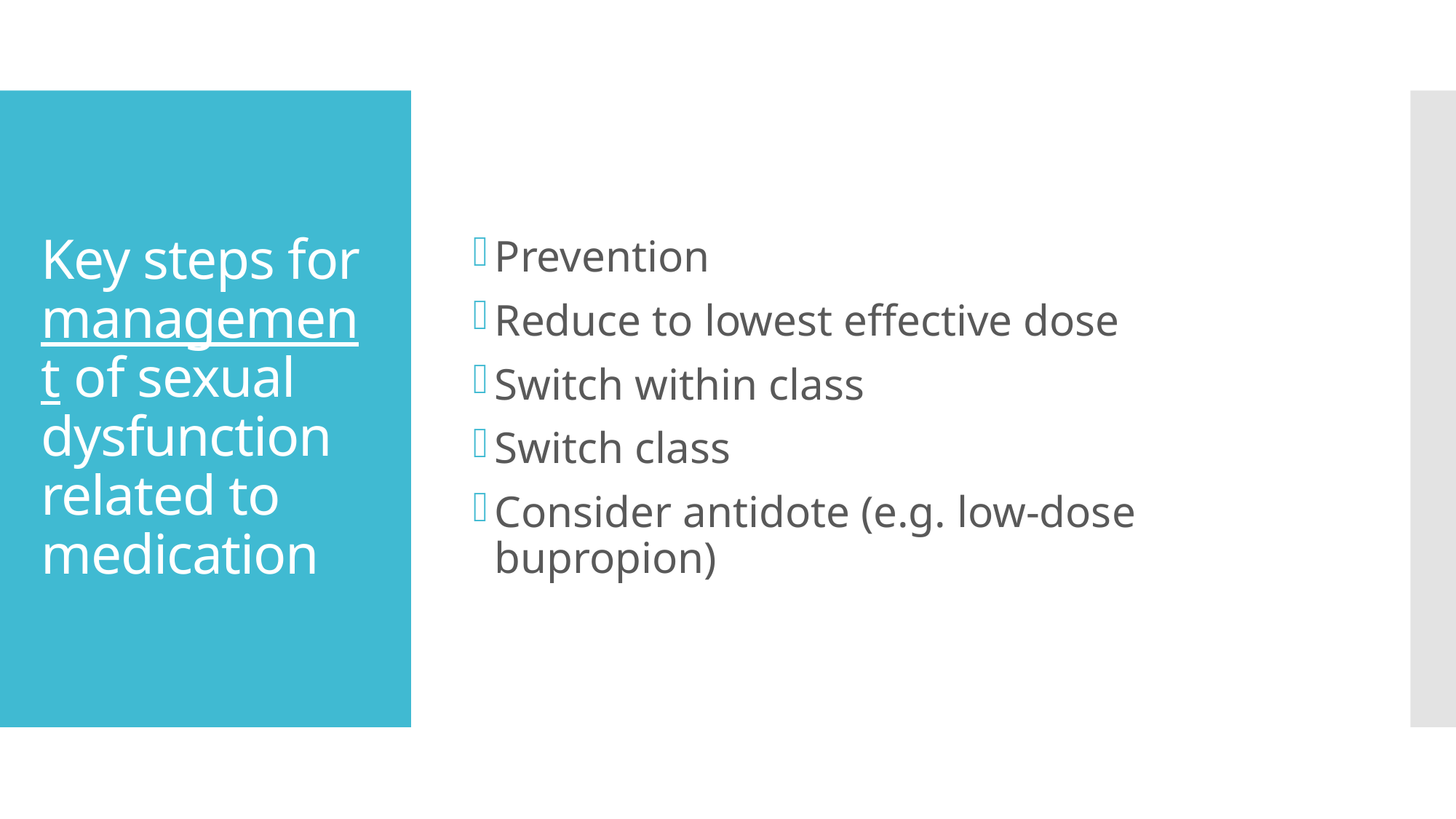

Prevention
Reduce to lowest effective dose
Switch within class
Switch class
Consider antidote (e.g. low-dose bupropion)
# Key steps for management of sexual dysfunction related to medication

## Slide 24
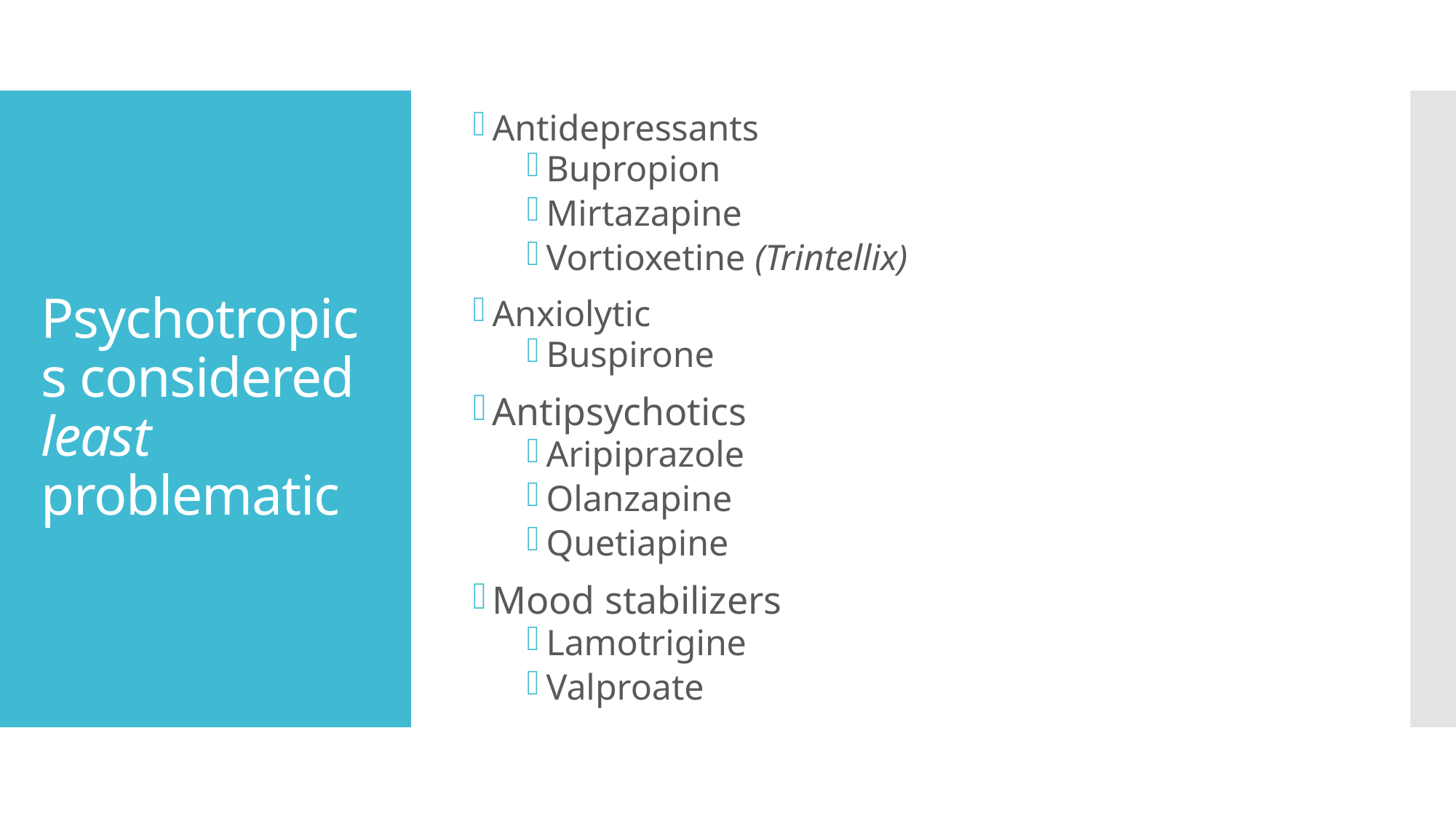

Antidepressants
Bupropion
Mirtazapine
Vortioxetine (Trintellix)
Anxiolytic
Buspirone
Antipsychotics
Aripiprazole
Olanzapine
Quetiapine
Mood stabilizers
Lamotrigine
Valproate
# Psychotropics considered least problematic

## Slide 25
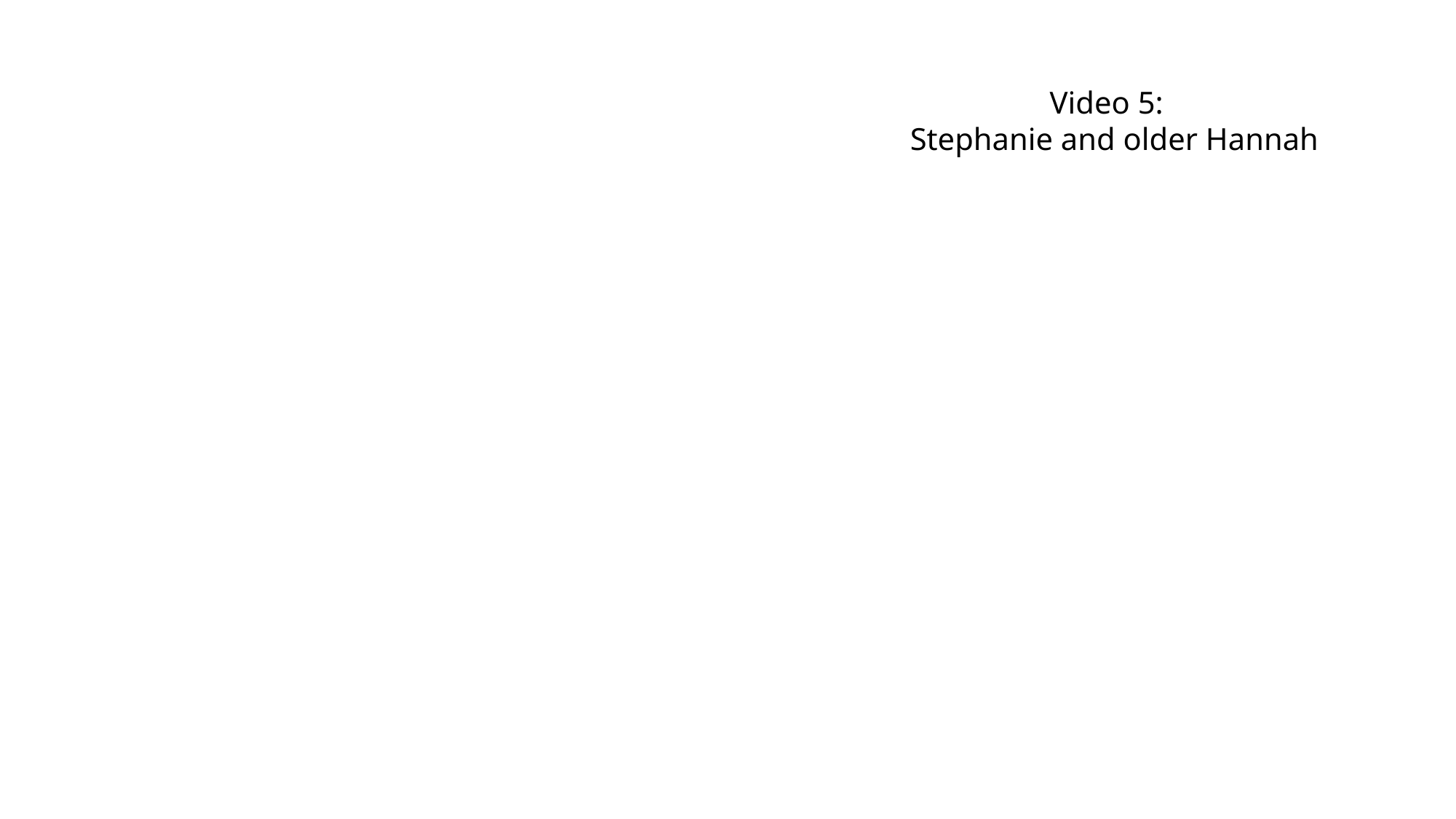

Video 5:
Stephanie and older Hannah

## Slide 26
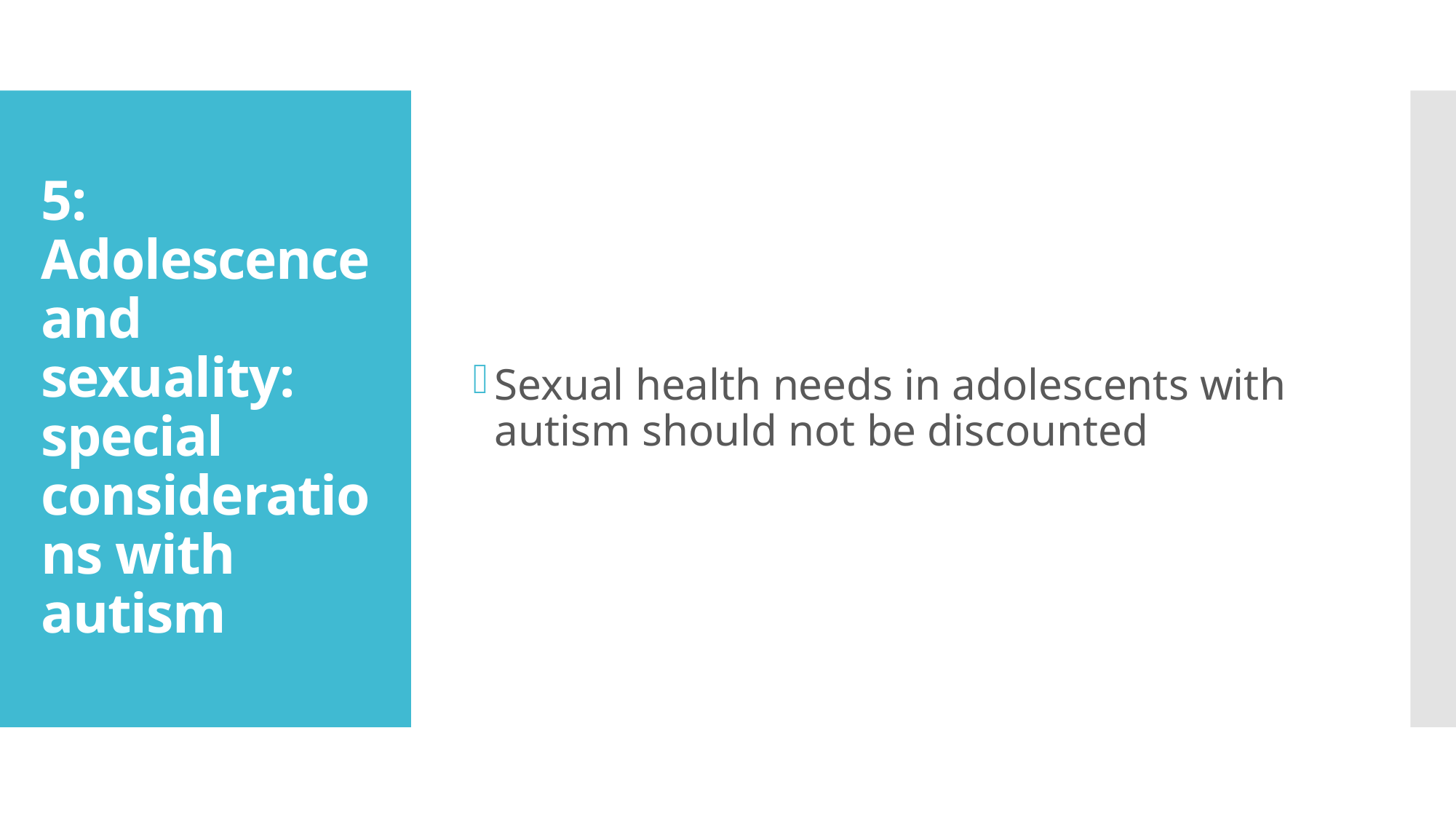

Sexual health needs in adolescents with autism should not be discounted
# 5: Adolescence and sexuality: special considerations with autism

## Slide 27
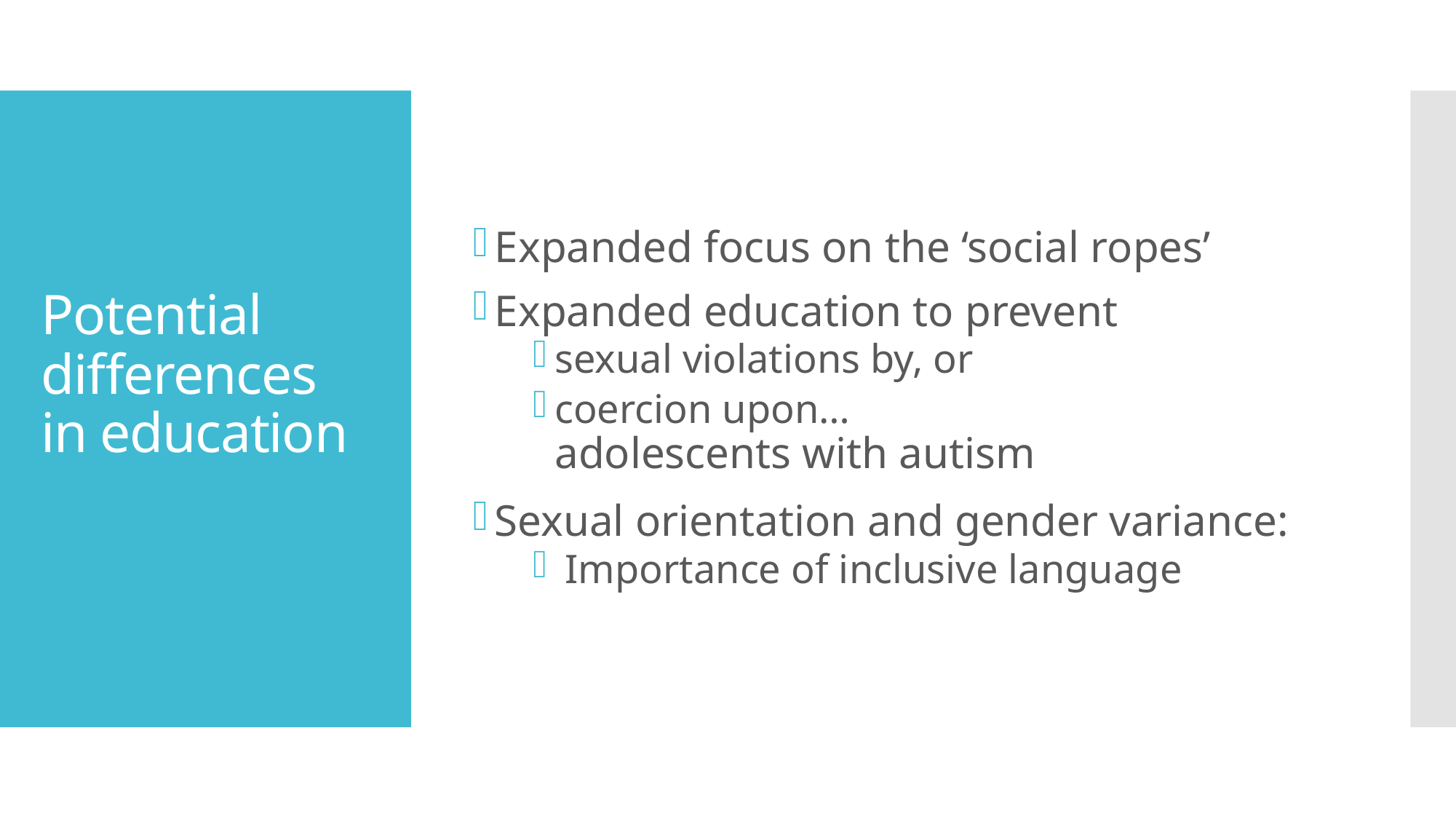

Expanded focus on the ‘social ropes’
Expanded education to prevent
sexual violations by, or
coercion upon…
adolescents with autism
Sexual orientation and gender variance:
 Importance of inclusive language
# Potential differences in education

## Slide 28
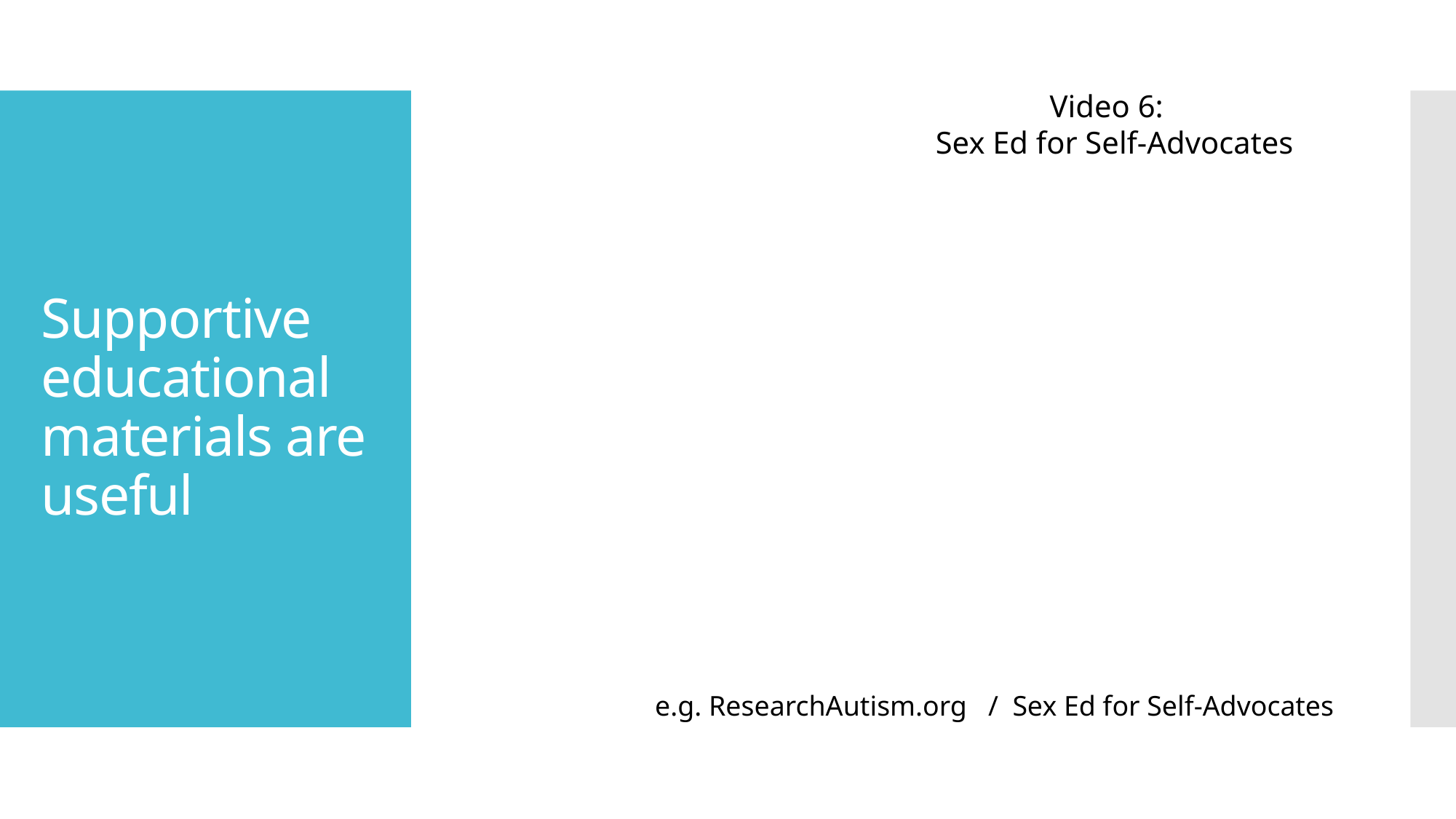

Video 6:
Sex Ed for Self-Advocates
# Supportive educational materials are useful
e.g. ResearchAutism.org / Sex Ed for Self-Advocates
