# Supplementary material for: Sexual Health in Child and Adolescent Psychiatry: Multi-Site Implementation Through Synchronized Videoconferencing of an Educational Resource Using Standardized Patients
Source: Front Psychiatry. 2020 Nov 17;11:593101. doi: 10.3389/fpsyt.2020.593101 (PMC7716796; doi:10.3389/fpsyt.2020.593101)
Supplement: Supplementary file 7 [file Data_Sheet_1.docx]

**SUPPLEMENTARY MATERIAL**

**I. Facilitator’s Guide / Handout**

***Didactic 1.
Sexual health and sexual development: why it matters and what is normal in childhood***

- **Focus:**
  - Sexuality in the pediatric population
  - Sexual health and normal sexual development
  - Why you must and how you can support sexual health, as a child and adolescent psychiatrist
- **Sexuality** (definition by WHO 2006)
  - A central aspect of being human throughout life encompass(ing) sex, gender identity and roles, sexual orientation, eroticism, pleasure, intimacy, and reproduction
  - Sexuality is experienced and expressed in thoughts, fantasies, desires, beliefs, attitudes, values, behaviors, practices, roles, and relationships
  - While sexuality can include all these dimensions, not all are always expressed or experienced
- **Sexual health** (definition by WHO 2006)
  - A state of physical, emotional, mental and social well-being in relation to sexuality; it is not merely the absence of disease, dysfunction or infirmity. Sexual health requires a positive and respectful approach to sexuality and sexual relationships, as well as the possibility of having pleasurable and safe sexual experiences, free of coercion, discrimination and violence
- How to ensure patients are sexually healthy:
  - Given the definitions, easy to understand how sexuality is a component of ALL humans’ lives
  - Address, assess, support, and treat sexual development and sexual health
- WHY sexual health matters in medical practice
  - Sexual health and sexuality are both a part of and related to overall health
  - You may do harm by not addressing it in your patient interactions
- Sexual health is positively, bidirectionally correlated to:
  - General health (physical, mental, and social/economic)
- Sexual health is positively related to:
  - At the individual level:
    - Physical and mental health
    - Longer life
    - Accomplishment of goals
    - Overall life satisfaction
  - At the community level:
    - Increased economic stability
    - Increased education and employment
    - Improved relationships

References:

The Surgeon General’s Call to Action to Promote Sexual Health and Responsible Sexual Behavior. Office of the Surgeon General (US); 2013

Hogben M., Ford J, et al. A systematic review of sexual health interventions for adults, narrative evidence. J Sex Res. 2015; 52; 444-469

Becansen J. Ford J., Hogben M. Sexual health interventions- a meta-analysis. J Sex Res. 2015; 52: 433-443

Ford J., Ivankovich M., Douglas M., et al. The need to promote sexual health in America: a new vision for public health action. Sex Transmit Dis. 2017; 44 (10): 579-585

Schwartzenruber A., Zenilman J. A national strategy to improve sexual health. JAMA. 2010; 304: 1005-1006


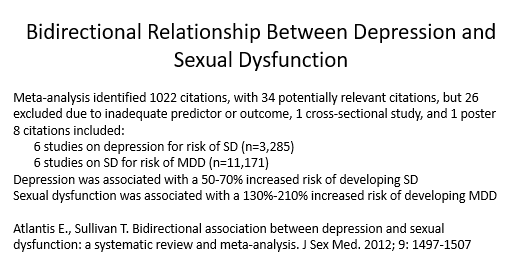


- Mental health and sexual health
  - Mental health and sexual health are dependent upon each other, making it crucial to address sexual health to support mental health treatment efforts
  - Depression is associated with 50-70% increased risk of sexual dysfunction (SD)
  - BUT – SD is associated with a 130-210% increased risk for depression!

Reference:

Atlantis E. and Sullivan T. bidirectional association between depression and sexual dysfunction. A systematic review and meta-anaylsis. J Sex Med 2012;9:1497-1507

- Physicians have a duty to address sexual health since
  - Psychotropics can cause significant sexual side effects: that is, treatments provided by physicians may be a cause of (unintentional) harm
  - Therefore, to mitigate this affect, assessment for iatrogenic SD is crucial
- Physicians are poised to decrease stigma
  - Stigma and secrecy around sexuality contribute to adverse sexual health outcomes
  - The two ways to decrease stigma are
    - Education – a proven way to improve sexual health outcomes
    - Normalization - frequently and neutrally addressing the topic
- Studies demonstrate adult patients want their physicians to address sexual health. While there is no good research examining the desires of the adolescent population to have their physician address the topic, evidence from childhood education communities would suggest that while adolescents may be more embarrassed about discussing the topic, they may also be more eager for education
  - The practitioner must initiate the discussion to prevent placing burden on the patient, who may be reluctant due to the stigma around sexuality

References:

Marwick C. Survey says patients expect little physician help on sex. JAMA. 1999: 281; 2173-2174

Janelle M, Sobecki M, et al. what we don’t talk about when we don’t talk about sex: results of a national survey of US Obstetricians/ gynecologists. 2012: 9: 1285-1294

Plaut T, Pai Lui M, et al. Discussing sexual health with your doctor. MAHEC Online J of Res. 2015: 2: 1-9

- Below is an overview of childhood sexual development, physical, behavioral, and safety considerations. However, the emphasis of this education should be on sexual health as a whole and it is crucial to discuss the positives as well as the negatives
  - Basic overview of childhood sexual behavioral development
    - <https://www.nctsn.org/sites/default/files/resources/sexual_development_and_behavior_in_children.pdf>
  - Basic education and safety education points
    - <https://www.nctsn.org/sites/default/files/resources/sexual_development_and_behavior_in_children.pdf>
- How to use this information in your work
  - Make sure you ask about sexual development in the initial clinical encounter
  - Normalize the topic by phrasing the introduction as “we ask all patients about sexual health and development”
  - The discussion is with the parents until child is pubertal, then the discussion can be had with the child alone IF
    - The child indicates they would prefer that
    - AND your practicing guidelines permit
  - Ask how they feel (emotionally) about their child’s/their own sexual development
  - Clarify what they expect
  - Provide basic education to support or correct their understanding
  - It is important to
    - Cover all topics, at least briefly
    - Provide parents or patients with
      - What is normal at each age
      - What to expect at the next stage of development, in advance
      - NOTE: education regarding puberty should begin in childhood, in advance of adolescence, around ages 7-8
  - Recommend parents regularly talk about sexual health with their children.
  - Parents should:
    - Know research shows education is correlated with improved sexual health
    - Talk with their child about sexual health
    - Start early and remember sexual health is not just about “sex”
    - Use real names for body parts and processes
    - Repeat the conversations, building on the information gradually and in an age appropriate manner
    - Answer any questions that come up honestly and with fact-based information. If they do not know an answer, they should say so and let the child know they will look it up and then discuss it

***Didactic 2.***

***Talking about sexual development in children with autism***

- **Focus:**
  - Sexual health needs are universal
  - Key differences in sexual health educational requirements for children with autism
- Everyone must be educated to develop and achieve sexual health
- For children with autism, most information and educational approaches are the same
  - Some key differences, to understand as a practitioner and for parents
    - Although the methods of delivering the information may be slightly different for children with autism, the information is the same
    - Change can be difficult for children with autism, and developmental changes may be frightening if they do not know what to expect
    - There may be a need to explain all sexual health and development concepts, as well as puberty, concretely and explicitly, with consistent and direct vocabulary
    - Books, websites, diagrams, written material, and demonstrations, can be particularly helpful
  - There are great resources geared to this population:

References:

The Growing Up Book for Boys: What Boys on the Autism Spectrum Need to Know! Davida Hartman- Margaret. Jessica Kingsley Publishers, 2015

What’s Happening to Ellie? A Book About Puberty for Girls and Young Women with Autism and Related Conditions Kate Reynolds and Jonathon Powell. Jessica Kingsley Publishers 2015

- As a practitioner, it is recommended to
  - Cover all topics, at least briefly
  - Discuss what is normal at each age
  - Discuss what to expect at the next stage of development, in advance
- With this population, using visual aids as well as social stories can be helpful.
  - Menstrual products and how to use them

<https://researchautism.org/sex-ed-guide-puberty/#physicalchanges> <https://vkc.mc.vanderbilt.edu/healthybodies/files/HealthyBodiesAppendix-Girls.pdf>

Additional Considerations

- There may be a need for additional education regarding the social components of sexual health
  - Children with autism may benefit from explicit explanations of what they CAN do, as well as what they CAN’T do
    - For example:
      - Teaching when and how/where they may hug or kiss a friend and when not to
      - Where (the physical locations) they may touch their genitals and where they may not

<https://vkc.mc.vanderbilt.edu/healthybodies/files/HealthyBodiesAppendix-Boys.pdf>

- - They may require additional education in public vs private behaviors
    - Children with autism may inadvertently engage in inappropriate behaviors
      - This may lead to serious legal repercussions, up to and including being labeled and registered as sex offenders
      - Detailed and concrete education about the social components can help prevent these incidents while also supporting the child’s social development

Resources

Books:

The Growing Up Book for Boys: What Boys on the Autism Spectrum Need to Know! - Print, available on Amazon

The Growing Up Guide for Girls: What Girls on the Autism Spectrum Need to Know! - Print, available on Amazon

What’s Happening to Ellie? A book about puberty for girls and young women with autism and related conditions (Sexuality and Safety with Tom and Ellie) - Print, available on Amazon

What’s Happening to Tom? A book about puberty for boys and young men with autism and related conditions (Sexuality and Safety with Tom and Ellie) - Print, available on Amazon

Taking Care of Myself: A Hygiene, Puberty and Personal Curriculum for Young People with Autism - Print, available on Amazon

Websites:

Healthy Bodies Toolkit - Parent’s Guide to Puberty for Boys and Girls with Disabilities from Vanderbilt Kennedy Center. Great appendices with social stories and other visual guides.

<https://vkc.mc.vanderbilt.edu/healthybodies/files/HealthyBodies-Boys-web.pdf>

Autismhelp.info - Online guide to sexuality, puberty, and hygiene for parents. <https://www.autismhelp.info/teen-years/teen-years-sexuality-puberty-hygiene>

Autism.org.uk - Online guide to sex education and puberty for parents. Some UK-specific language, but good concepts and explanations.

<https://www.autism.org.uk/about/communication/sex-education.aspx>

***Didactic 3.***

***Normal sexual development in adolescence, pornography, and ‘porn literacy’***

- **Focus:**
  - Sexual health and normal sexual development in adolescents
  - Key components of sexual health education for adolescents and their adults
  - The need to address pornography

Adolescent Sexual Health and Development

- Prior to adolescence, expanded sexual health education is needed with a focus on puberty and developing sexuality
- Research supports valid and comprehensive sexual health education is associated with multiple beneficial outcomes, including
  - Delayed engagement in sexual activity
  - Decreased risk taking behavior
  - Improved use of protection/ safety measures
  - Decreased rates of teen pregnancy and STI transmission
  - Increased rates of satisfaction with first encounter and ongoing

Reference:

Hildie L, Daniel T, et al. Development of contextually relevant sexuality education: Lessons from a comprehensive review of adolescent sexuality education across cultures. Internat J of Environ Res and Pub Health. 2019: 16; 621

Adolescent sexual development charts are available at:
https://www.michigan.gov/documents/mdch/Adolescent_Sexual_Development_292756_7.pdf)

- The cognitive, emotional, and social components of sexuality that teens need to understand and incorporate to be sexually healthy are as important as the physical milestones

Outline of the physical, cognitive, emotional, and social considerations to support adolescent sexual health for practitioners to know or share with parents or patients are available at: <https://advocatesforyouth.org/resources/health-information/parents-16/>

- Approaches to and values around sexuality vary widely. Practitioners must respect and support those values of your patient or patient’s family in so far as they are compatible with health
- Parents should clearly articulate their family and religious values regarding sexual activity and intercourse. Parents can acknowledge sex is pleasurable, while also emphasizing the adolescent should wait to initiate sexual activity in alliance with their family values
- Use inclusive language that recognizes that some youth may be of different gender identity, or orientation than may have let on, or they may be still exploring these constructs
- Reinforce adolescents’ ability to make decisions while providing information to base the decisions on
- Acknowledge adolescents are sexual (not the same as sexually active) and help them understand the options and consequences of sexual expression
- Express we all have a variety of options for experiencing intimacy and expressing love
  - Parents should acknowledge adolescents have many future life options (Ex: some may marry and/or parent while others may remain single and/or childless)
- Understand adolescents will develop or may already have an understanding of their own sexual orientation (which is distinct from their sexual behavior)
- Reinforce they can choose to express their sexuality in ways that may or may not include shared sexual behaviors
- Parents should discuss the factors (including age, mutual consent, protection, contraceptive use, love, intimacy, etc.) that they and their adolescent believe should be a part of decisions about various levels of sexual engagement
- Reinforce adolescents have the capacity and need to learn about intimate, loving, long-term relationships, and doing so is a process
  - Educate them to recognize the components of healthy and unhealthy relationships, physical and emotional safety, what exploitive behavior is and why it is unhealthy and (in some cases) illegal
  - Help them identify various physical and verbal responses and behavioral plans to avoid or escape sexual situations that make them feel uncomfortable
- Educate about and reinforce that, as an adult, you are open to discussing, the emotional components of attraction, relationships, and relating sexually
- Educate about the physical realities of
  - Pleasure as a component of sexuality
  - But also pregnancy, HIV and other STI’s,
  - And what safer sex measures are available and how to use them (“safer sex” is a phrase used to underscore the reality that no protective measure is 100% safe)
  - Emphasize the need for barrier method protection
- Parents should discuss teens’ options
  - Should unprotected intercourse occur — including emergency contraception and STI testing and treatment
  - Should pregnancy occur, including abortion, parenting, and adoption
- Educate adolescents in sexual social-media literacy; that is- to understand and recognize
  - The impact various media have on cultural views about sex
  - The way sexual activity in real life differs from the various media representations
  - How to be sexually safe in a digital world, and online

Pornography and Porn Literacy

- Pornography may be a difficult topic for parents. Parents may believe, to some degree correctly, that pornography is more available now
- This makes pornography an important topic to address to support patients’ sexual health, both with the parents and/ or with adolescent patients
- The average age of first porn viewing, intentional or not, is likely around 11-13, though can be earlier. NOTE: studies on this are all problematic, with some widely quoted data being based on shoddy research, but, it is agreed upon that first exposure is relatively early and both early childhood educators and high school educators agree, based on experience, that pretty much all children they deal with have encountered some form of pornography by high school. But the most widely quoted studies are below

Reference:

A trend analysis of U.S. adolescents’ intentional pornography exposure on the internet, 2000-2010. Rothman E and Mitchell K. Boston University School of Public Health.

- Anticipatory education is important. Parents should start the conversation early, before their child is a teen
- Parents should help their children achieve “porn literacy”
- Porn Literacy
  - - - Understanding what pornography is and how to ethically incorporate it into your life if you choose to use it
      - Similar to media literacy
  - Provide education about how pornography is not representative of real sexual activity, but that it is like other movies, designed for recreational purposes and complete with fantastical plots (or lack thereof), special effects and editing
  - As a product designed to entertain, the representations of bodies, gender roles, and sexual encounters are highly stylized
  - This knowledge, together with education around how porn differs from real life engagement, helps ensure children are not
- Learning only from pornography
- Anxious if/ when their eventual sexual encounters are not like those in pornography
  - Provide education about the concept of ethical porn: that is the consideration of whether the actors in the porn were of legal age and willing participants

Resources:

Adolescent sexual development and understanding pornography and porn literacy

Websites (articles and multi-ed/resource sites):

- <http://amaze.org/>
- <https://www.youtube.com/watch?v=GdB2rmGqqNU#action=share>
- <https://www.youtube.com/watch?v=GdB2rmGqqNU#action=share>
- <http://givethetalk.com>
- <http://thenovusproject.org/>
- APA sexuality education - <https://www.healthychildren.org/>
- <https://www.Seicus.org>
- <https://brightfutures.aap.org/Bright%20Futures%20Documents/BF4_HealthySexuality.pdf>
- <https://www.nctsn.org/sites/default/files/resources/sexual_development_and_behavior_in_children.pdf>
- <https://www.ncbi.nlm.nih.gov/pmc/articles/PMC4477452/>
- <https://www.michigan.gov/documents/mdch/Adolescent_Sexual_Development_292756_7.pdf>
- <https://pedsinreview.aappublications.org/content/34/1/29>
- <https://www.karger.com/Article/FullText/367857>
- https://www.sciencedirect.com/science/article/pii/S0747563219300299
- <https://aifs.gov.au/publications/effects-pornography-children-and-young-people-snapshot>
- <https://advocatesforyouth.org/resources/health-information/parents-16/>
- Article:<https://www.google.com/amp/s/www.yahoo.com/amphtml/lifestyle/wish-parents-talked-sex-170608203.html>

Books

For children

- It's So Amazing - Robie Harris (5-10)
- Its' Perfectly Normal - Robie Harris (ages 8ish-10-ish)
- What’s Happening to Me? – Peter Mayle (9-12)
- What's Happening to My Body (1 for boys and 1 for girls) - Lynda Madaras and Area Madaras (10-11-15)
- Sex: A Book for Teens – Nikol Hassler (16+)
- The Naked Truth About Sex: A Guide to Intelligent Sexual Choices for Teenagers and Twentysomethings - Roger Libby

For Parents

- Sex and Sensibility: The Thinking Parent's Guide to Talking Sense About Sex - Deborah Hoffman
- Everything you never wanted your kids to know about sex but were afraid they’d ask – Justin Richardson and Mark Schuster
- Educate 2 empower - Preparing yourself for your child’s sex education

***Didactic 4.
The management of medication-induced sexual dysfunction***

- **Focus:**
  - The medications that can induce SD
  - The importance of addressing iatrogenic SD
  - Management strategies for addressing SD
- Sexual dysfunction associated with medical illness and medications is a fairly frequent and complicated topic
  - For example: in a patient with MDD treated with an SSRI, reporting SD, the SD may be due to (some may be less likely in the pediatric population):
  - The depression/ primary illness
  - A side effect of the medication
  - A concomitant medical illness/ non-primary illness
  - A side effect of a medication used to treat the concomitant illness
  - Secondary to substance use
  - Primary SD
  - Associated with relationship difficulties
  - A combination of more than one or all of these
    - More recent research supports that stages of the sexual response cycle likely influence each other in a circular, not linear fashion (Basson Circular Sexual Response Cycle)
    - With consideration to SD of arousal: anywhere erectile dysfunction is cited as an outcome, consider the possibility of potential arousal dysfunction in women as well. Women have the same amount of erectile tissue within the clitoral complex, which, physiologically, is activated in the same way
- Medication Induced Sexual Dysfunction is its own diagnosis

| The Diagnostic Criteria for Substance/ Medication Induced Sexual Dysfunction  (From The Diagnostic and Statistical Manual of Mental Disorders, Fifth Edition - American Psychiatric Association) |
| --- |
| 1. Clinically significant disturbance in sexual function is predominant in clinical picture 2. There is evidence from the history, physical examination, or laboratory findings of both (1) and (2):    1. (1) The symptoms in Criterion A developed during or soon after substance intoxication or withdrawal or after exposure to medication    2. (2) The involved substance/ medication is capable of producing the symptoms in Criterion A 3. The disturbance is not better explained by sexual dysfunction that is not substance/ medication-induced. Such evidence of an independent sexual dysfunction could include the following: the symptoms precede the onset of the substance/medication use; the symptoms persist for a substantial period of time (e.g. about 1 month) after the cessation of acute withdrawal or severe intoxication; or there is other evidence suggesting the existence of an independent non-substance/ medication induced sexual dysfunction (e.g. a history of recurrent non-substance/ medication related episodes) 4. The disturbance does not occur exclusively during the course of a delirium 5. The disturbance causes clinically significant distress in the individual |

- Psychotropics are notorious for SD side effects
- Both from the medication and, potentially, from/after discontinuation.
  - - Multiple reports of SD not resolving even after discontinuation of the medication
    - Abrupt cessation of SSRI is one of the largest contributors to new onset PGAD (Persistent Genital Arousal Disorder)
- These SD are problematic
  - Sexual side effects are a significant cause of medication non-compliance
  - For the pediatric population, patients may require long term medication for treatment, making it necessary to address the topic early and ensure it does not become an issue that interferes with their willingness to engage
  - With adolescents, there is potential interference with critical sexual development and exploration, causing them to feel or actually be alienated from their peers
- List of Groups of Medications Commonly Used in Psychiatry and Associated Sexual Dysfunctions
  - Adapted from table 23-1 (Types of Sexual dysfunction associated with selected groups of medications and effects of substance of abuse) from The Textbook of Clinical and Sexual Medicine. Editors: Waguih, William, and Ishak. Springer International Publishing AG 2017)

| **Medications** | **Sexual Dysfunction(s)** |
| --- | --- |
| Antidepressants   - SD likely in 40-50% of patients, - Provided careful evaluation | Changes in libido (mostly decreased)  Erectile dysfunction  Changes in lubrication  Delayed ejaculation/ orgasm (SSRI’s, clomipramine)  Anorgasmia (SSRI’s, clomipramine)  Priapism (trazodone and others)  Clitoral engorgement  Painful ejaculation (TCAs)  Penile or vaginal anesthesia |
| Antipsychotics   - SD likely in 50% of patients - significantly less frequent with atypical agents | Decreased libido  Erectile dysfunction  Delayed ejaculation  Priapism  Retrograde ejaculation (thiothixene)  Painful ejaculation  Amenorrhea  Galactorrhea |
| Anxiolytics | Decreased libido  Delayed or inhibited ejaculation  Sexual disinhibition (?) |
| Mood stabilizers/anticonvulsants | Decreased libido  Ejaculatory dysfunction  Anorgasmia/ ejaculatory failure  Decreased orgasm satisfaction  Loss of orgasm sensation  Unpleasant feeling upon touching the genitalia or erogenous zones |

- Evidence supporting which meds are better with regard to SD is limited, but one meta-analysis of the existing literature provided the below list
  - List of medications in order of most to least impact (rates ranging from 80.3% to 25.8%)

Adapted from: Serreti A. Chiesa A. Treatment-emergent sexual dysfunction related to antidepressants: a meta-analysis. J Clin Psychopharmacol. 2009;29:259-66

- - - sertraline
    - venlafaxine
    - citalopram
    - paroxetine
    - fluoxetine
    - imipramine
    - phenelzine
    - duloxetine
    - escitalopram
    - fluvoxamine
    - NOTE: no significant difference was found compared to placebo for agomelatine, amineptine, bupropion, moclobemide, mirtazapine, nefazedone
- In summary: those medications that are considered least problematic are:
  - For Depression
    - Bupropion, mirtazapine, nefazodone, vortioxetine
  - For Anxiety Disorders
    - Buspirone

Evaluation:

- Unfortunately, there are no diagnostic laboratory tests or diagnostic instruments for measuring human sexuality as a whole. But, there are a number of good instruments to measure discrete phases or specific aspects of sexual function and dysfunction

| Instruments to Measure Sexual Dysfunction (Most Commonly Used, Not Complete)  Adapted from table 23-3: Selected instruments to measure sexual dysfunction associated with medications. The Textbook of Clinical and Sexual Medicine. Editors: Waguih, William, and Ishak. Springer International Publishing AG 2017 |
| --- |
| - Arizona Sexual Experience Scale (ASEX) - Changes in Sexual Functioning Questionnaire (CSFQ) - Modified Rush Sexual Inventory (MRSI) - Psychotropic Related Sexual Function Questionnaire (PrSexDQ or SALSEX) - Sex Effects Scale (SexFx) - Derogatis Interview for Sexual Functioning (DISF/ DISF-SR) - International Index of Erectile Function (IIEF) |

- Whether or not scales are utilized, clinical discussion is crucial
- When conducting a clinical evaluation, start from more general open ended questions and progress to more specific inquiry, as patients may be reluctant to provide detail on their own

Management:

- The practitioner must initiate the initial discussion and frequent follow up about SD
- SD incidence is higher when actively asked for by investigators/ practitioners (58 vs 14% in one study and 41 vs 6% in another) likely because of embarrassment around discussing a stigmatized topic, so it is incumbent upon you to actively monitor for and ask about SD

References:

Montejo-Gonzales A, Llorca G, et al. SSRI induced sexual dysfunction: fluoxetine, paroxetine, sertraline, and fluvoxamine in a prospective, multicenter, and descriptive clinical study of 334 patients. J sex marital Th. 1997; 23:176-94

Landen M, Hogberg P, et al.Incidence of sexual side effects in refractory depression during treatment with citalopram or paroxetine. J Clin Psych. 2005;66:100-6

- When SD occurs, the management of SD “remains more of a clinical art than a science, and relies on combining weak evidence from literature with clinical skills, and improvisation based on the clinical picture, medication profile, and the patient’s interest and willingness” (The Textbook of Clinical Sexual Medicine)
- The element of creativity in management may be especially important for the adolescent population, given the fact that some of the “antidotes” are not approved for pediatric use
- Therefore, prevention may be of greater importance and, should there be SD to treat, trials of dosage changes or changes of medications may be the most effective strategies

| General Guidelines for A Management Strategy for Psychotropic Induced Sexual Dysfunction  NOTE: not all strategies are recommended with all patients  Adapted from table 23-4 (Management strategies for sexual dysfunctions associated with psychotropic medications) from The Textbook of Clinical and Sexual Medicine. Editors: Waguih, William, and Ishak. Springer International Publishing AG 2017 |
| --- |
| 1. Select a medication with a low incidence of sexual dysfunction, especially in sexually active patients and in patients on psychotropics for the first time 2. Encourage lifestyle changes or adjustments (diet, exercise, sleep hygiene, no substances of abuse) 3. Wait for spontaneous remission of sexual dysfunction or patients’ accommodation to it 4. Reduce medication to the lowest effective dose 5. Schedule sexual activity around dosing of medication 6. Switch to another medication from the same class with a lower frequency of SD 7. Use drug holidays 8. Use other medications to counter the effects of SDs 9. Use sex therapy, mindfulness, CBT, psychotherapy 10. Exercise (general and) before sexual activity 11. Use mechanical intervention |

- Management within Classes of Drugs
  - Antidepressants
- Start by trying med with low incidence of SD if you can
- Wait for spontaneous remission (requires a very good doctor patient relationship, and may lead to medication non-adherence, not a strongly recommended strategy)
- Schedule sexual activity around dosing of medications
- Reduce the dose to the minimum effective dose
- Switch to another medication in the same class with lower SD side effect profile
- Use antidotes
  - - PDE5 inhibitors –
      - They may be effective for both men and women reporting SSRI related SD, but current evidence supports using them for men and bupropion for women
- Bupropion - added in low dose, may be of some benefit for men and women
- Flibanserin - now approved for low desire in adult women, but must be used with care, as it is technically a failed SSRI
- Bremelanotide - now approved for low desire in adult women
- Use mechanical Intervention
  - - Vacuum pumps
    - EROS-CTD (essentially a vacuum pump for the clitoris, it is one of 2 FDA approved treatments for sexual dysfunction)
    - Vibrators – may help for men and women who feel a dulling of sexual or genital sensation on medications
- Acupuncture - one study demonstrated efficacy

Reference:

Khamba b, Aucoin M, et al. Efficacy of acupuncture treatment of sexual dysfunction secondary to antidepressants. J Altern Complement Med. 2013; 19: 862-9

- Antipsychotics
  - Try starting with a med with a lower incidence SD, if you can
    - Atypical are associated with a lower risk of SD, but some may also be more associated with increased prolactin, which may contribute to SD
    - Aripiprazole may have lowest incidence
    - Olanzapine
    - Quetiapine
  - Other management strategies are similar to those for antidepressants
- Mood stabilizers
  - Try starting with a med with lower incidence of SD, if you can
    - Lamotrigine
    - Valproic acid (but not in childbearing aged women)
    - Consider an atypical antipsychotic
  - Other management strategies are similar to those for antidepressants

Resources:

Chapter 23 The Textbook of Clinical and Sexual Medicine. Editors: Waguih, William, and Ishak. Springer International Publishing AG 2017

***Didactic 5.
Special sexuality considerations with autism during adolescence***

- **Focus:**
  - Key differences in sexual health educational requirements for adolescents with autism
  - Special sexual health considerations in adolescents with autism
- Often, sexuality is not considered in the context of those with disability. It is not uncommon for adults, including parents, to infantilize adolescents with autism (or with any disability) and to assume they will not be interested in sexual activity or relationships
- These assumptions and behaviors can hinder their sexual health
- Research shows most people with autism show interest in solitary or partnered sexual experiences, just as with neurotypical counterparts

References:

Byers ES, Nichols S, Voyer SD. Challenging stereotypes: sexual functioning of single adults with high functioning autism spectrum disorder. J Autism Dev Disord 2013; 43:2617–2627.

Dewinter J, Van Parys H, Vermeiren R, van Nieuwenhuizen C. Adolescent boys with an autism spectrum disorder and their experience of sexuality: an interpretative phenomenological analysis. Autism 2017; 21:75–82.

Dewinter J, Vermeiren R, Vanwesenbeeck I, et al. Sexuality in adolescent boys with autismspectrum disorder: self-reported behaviours and attitudes. J Autism Dev Disord 2015; 45:731–741.

Dewinter J, Vermeiren R, Vanwesenbeeck I, Van Nieuwenhuizen C. Adolescent boys with autism spectrum disorder growing up: follow-up of self-reported sexual experience. Eur Child Adolesc Psychiatry 2016; 25:969–978.

Byers ES, Nichols S, Voyer SD, Reilly G. Sexual well being of a community sample of high-functioning adults on the autism spectrum who have been in a romantic relationship. Autism2012; 17:418–433.

Strunz S, Schermuck C, Ballerstein S, et al. Romantic relationships and relationship satisfaction among adults with Asperger syndrome and high-functioning autism. J Clin psychol 2017; 73:113–125.

- For adolescents with autism the need for education around puberty and their developing sexuality is much the same as for the neurotypical population, and also should start in advance of adolescence
- There are some differences in the specifics that are important to know and/or share with patients or parents
  - The delivery method
  - Some expanded details
  - Increased focus on safety
  - Differences in population sexuality
- Adolescents with autism often require explicit and concrete education
- Again, education about things they CAN do, not just what they CAN’T do, is important
- In particular, adolescents with autism may require extremely detailed and concrete education around the “social ropes” and nuances of courtship, relationships, and sexuality
  - For example: explaining the detailed steps of how to ask someone out for a date and how to interpret and respond to different answers can be crucial for peer interactions and support their social, mental, and sexual health
    - This may sound like: “If you ask someone out on a date, and they tell you they have other plans, you may ask them out one more time. The next time, even if they again say they can’t go because of other plans, do not ask them out again.”
- The risk of unintended sexual violations and sexual coercion or predation upon this population is high. Concrete, explicit, and detailed education can help mitigate that risk
  - For example: discussing what coercive behavior is and how that can be at odds with verbal expression could be important
    - - This may sound like: “Even if a person says “I love you” while engaging in (the described coercive behavior) or demanding unwanted action, it is still coercion.”
  - Establish detailed plans for what to do in cases of coercion or victimization, just as with neurotypical teens
  - Detailed and concrete discussions of public and private behaviors also mitigate risk. Engaging in overt sexual behaviors in public could both put an adolescent at higher risk of being victimized while also putting them at risk of victimizing others
    - - For example: a private boundary must be clearly defined
      - This may sound like: “You should only masturbate when you are in private in the home.”
- Language and an approach that is inclusive of the wide variety of sexual identities and orientations is especially important with adolescents with autism. Data suggests sexual orientation is more varied in those with autism when compared to controls
  - In one study of ~700 people with autism, only 56% of ASD women indicated that they feel attracted to men compared with almost 90% of the female healthy controls. 22% of ASD women reported being bisexual, 6% homosexual, and 14% attracted neither to men nor women

Reference:

Dewinter J, De Graaf H, Begeer S. Sexual orientation, gender identity, and romantic relationships in adolescents and adults with autism spectrum disorder. J Autism Dev Disord 2017; 47:2927–2934

- Also, again, supportive educational materials and resources can be particularly useful for this population, and there are some excellent ones available
  - NOTE: Many of these listed resources are geared to parents or general sex educators, but are still useful for practitioners and to recommend to patients who want to learn on their own
  - For adolescents 15 years of age and up who are interested in learning on their own, we recommend the ResearchAutism.org guide to “Sex Ed for Self-Advocates”

Resources:

For adolescents:

- <https://researchautism.org/sex-ed-guide/>
- <http://www.iwannaknow.org/teens/sexualhealth/puberty.html>
- <https://kidshealth.org/en/kids/puberty.html>
- <https://www.plannedparenthood.org/learn>
- List of print resources found at: <https://researchautism.org/sex-ed-resources/#selfadvocateprintresources>

For parents/practitioners:

- <http://www.danya.com/autism_pdfs/STAR%20Charting%20the%20Course%20Parent%20Guide.pdf>
- <https://www.cigna.com/static/www-cigna-com/docs/individuals-families/bhs-autism-2017-september-handout.pdf>
- <https://www.plannedparenthood.org/planned-parenthood-massachusetts/local-training-education/parent-buzz-newsletter/parent-buzz-e-newsletters/sexuality-education-youth-autism-spectrum>
- <https://www.gvsu.edu/cms4/asset/64CB422A-ED08-43F0-F795CA9DE364B6BE/gerhardt_sexuality_ppt.pdf>
